# A suite of genome-engineered hepatic cells provides novel insights into the spatiotemporal metabolism of apolipoprotein B and apolipoprotein B–containing lipoprotein secretion

**DOI:** 10.1093/cvr/cvae121

**Published:** 2024-06-04

**Authors:** Amber Meurs, Klevis Ndoj, Marlene van den Berg, Goran Marinković, Matteo Tantucci, Tineke Veenendaal, Jan Albert Kuivenhoven, Judith Klumperman, Noam Zelcer

**Affiliations:** Department of Medical Biochemistry, Amsterdam UMC, Amsterdam Gastroenterology Endocrinology Metabolism and Amsterdam Cardiovascular Sciences, University of Amsterdam, Meibergdreef 9, 1105AZ Amsterdam, The Netherlands; Department of Medical Biochemistry, Amsterdam UMC, Amsterdam Gastroenterology Endocrinology Metabolism and Amsterdam Cardiovascular Sciences, University of Amsterdam, Meibergdreef 9, 1105AZ Amsterdam, The Netherlands; Department of Medical Biochemistry, Amsterdam UMC, Amsterdam Gastroenterology Endocrinology Metabolism and Amsterdam Cardiovascular Sciences, University of Amsterdam, Meibergdreef 9, 1105AZ Amsterdam, The Netherlands; Department of Medical Biochemistry, Amsterdam UMC, Amsterdam Gastroenterology Endocrinology Metabolism and Amsterdam Cardiovascular Sciences, University of Amsterdam, Meibergdreef 9, 1105AZ Amsterdam, The Netherlands; Center for Molecular Medicine—Cell Biology, University Medical Center Utrecht, University of Utrecht, Heidelberglaan 100, 3584CX Utrecht, The Netherlands; Center for Molecular Medicine—Cell Biology, University Medical Center Utrecht, University of Utrecht, Heidelberglaan 100, 3584CX Utrecht, The Netherlands; Department of Pediatrics, University Medical Center Groningen, University of Groningen, Antonius Deusinglaan 1, 9713 AV Groningen, The Netherlands; Center for Molecular Medicine—Cell Biology, University Medical Center Utrecht, University of Utrecht, Heidelberglaan 100, 3584CX Utrecht, The Netherlands; Department of Medical Biochemistry, Amsterdam UMC, Amsterdam Gastroenterology Endocrinology Metabolism and Amsterdam Cardiovascular Sciences, University of Amsterdam, Meibergdreef 9, 1105AZ Amsterdam, The Netherlands

**Keywords:** APOB, Hepatocytes, MASLD, ERAD, SYVN1, HRD1, VLDL, Lipoprotein metabolism

## Abstract

**Aims:**

Apolipoprotein B (APOB)-containing very LDL (VLDL) production, secretion, and clearance by hepatocytes is a central determinant of hepatic and circulating lipid levels. Impairment of any of the aforementioned processes is associated with the development of multiple diseases. Despite the discovery of genes and processes that govern hepatic VLDL metabolism, our understanding of the different mechanistic steps involved is far from complete. An impediment to these studies is the lack of tractable hepatocyte-based systems to interrogate and follow APOB in cells, which the current study addresses.

**Methods and results:**

To facilitate the cellular study of VLDL metabolism, we generated human hepatic HepG2 and Huh-7 cell lines in which CRISPR/Cas9-based genome engineering was used to introduce the fluorescent protein mNeonGreen into the *APOB* gene locus. This results in the production of APOB_100_-mNeon that localizes predominantly to the endoplasmic reticulum (ER) and Golgi by immunofluorescence and electron microscopy imaging. The production and secretion of APOB_100_-mNeon can be quantitatively followed in medium over time and results in the production of lipoproteins that are taken up via the LDL receptor pathway. Importantly, the production and secretion of APOB-mNeon is sensitive to established pharmacological and physiological treatments and to genetic modifiers known to influence VLDL production in humans. As a showcase, we used HepG2-APOB^mNeon^ cells to interrogate ER-associated degradation of APOB. The use of a dedicated sgRNA library targeting all established membrane-associated ER-resident E3 ubiquitin ligases led to the identification of SYNV1 as the E3 responsible for the degradation of poorly lipidated APOB in HepG2 cells.

**Conclusions:**

In summary, the engineered cells reported here allow the study of hepatic VLDL assembly and secretion and facilitate spatiotemporal interrogation induced by pharmacologic and genetic perturbations.

## Graphical Abstract

**Graphical Abstract:**
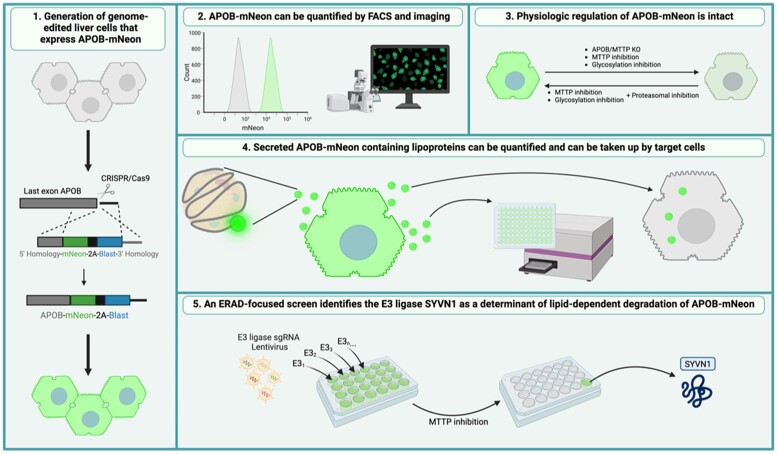

**Time of primary review: 51 days**

## 1. Introduction

The liver serves as the central organ governing systematic lipid metabolism, as it continuously produces, secretes, and clears lipids from circulation. Accumulation of triglycerides and cholesterol in the liver is a hallmark of metabolic dysfunction–associated steatotic liver disease (MASLD)^1^ and is viewed as the hepatic manifestation of the metabolic syndrome.^2^ As such, the development of MASLD shares many common risk factors with those associated with developing the metabolic syndrome, including, among others, a high-caloric diet, a sedentary lifestyle, and insulin resistance. Moreover, dysregulation of hepatic lipid and lipoprotein metabolism is intimately linked with the development of atherosclerotic cardiovascular disease.^3,4^ Following carbohydrate intake, insulin stimulates cholesterol and triglyceride biosynthesis in hepatocytes,^5^ leading subsequently to the production and secretion of very low-density lipoprotein (VLDL) to supply these lipids to peripheral tissues.^3^ Apolipoprotein B_100_ (APOB), the structural protein component of VLDL, is required for binding to membrane receptors such as the LDL receptor (LDLR) and facilitates the subsequent endocytosis of VLDL.^6^

APOB is highly expressed in hepatocytes and, with its 4563 amino acids, ranks as one of the largest proteins in the human proteome. APOB is continuously synthesized and, unless adequately lipidated by microsomal triglyceride transfer protein (MTP, encoded by the *MTTP* gene) to form a nascent particle in the endoplasmic reticulum (ER), is subject to ER-associated degradation (ERAD).^7–9^ The rate of VLDL production by hepatocytes is a primary determinant of plasma VLDL levels.^10^ In line with this, therapeutic targeting or mutations in *APOB* or *MTTP* are associated with reduced atherosclerotic cardiovascular disease but come at the expense of increased risk for fatty liver development.^11–13^ Despite four decades of research and numerous recent studies in mice, our understanding of the mechanisms and genes underlying the production, degradation, lipidation, and intracellular trafficking of VLDL is still incomplete.^14,15^

The lack of experimentally tractable hepatic human cell systems is an impediment to studying cellular APOB metabolism. The large size of APOB impedes straightforward cellular, biochemical, and genetic strategies in cells.^16^ Nevertheless, COS-7 cells over-expressing green fluorescent protein (GFP)-tagged APOB constructs were shown to be useful for studying cellular APOB metabolism.^17^ However, this cell system relies on non-physiological over-expression and regulation of APOB and on non-human cells in which APOB is not physiologically expressed. Recently, a Zebrafish model genetically engineered to express *apoB* fused to luciferase was reported.^18^ This elegant model allows *in vivo* kinetic studies of apoB distribution and lifetime and supports genetic interrogation of apoB metabolism in a relevant physiologic setting. Nevertheless, the availability of a tractable human hepatic cell model would greatly facilitate a robust interrogation of APOB metabolism and inform the mechanisms underlying development of MASLD in humans. Hence, stimulated by the strategies above, we used CRISPR/Cas9-mediated genome engineering to develop a suite of human hepatic cell lines (HepG2 and Huh-7 cells) in which the *APOB* alleles are endogenously tagged with the bright fluorescent protein mNeonGreen. Here, we report that these cells produce and secrete APOB-mNeon-containing lipoproteins that are subject to known physiologic, pharmacologic, and genetic regulators. Furthermore, using these cells, we identify SYVN1 as the E3 ubiquitin ligase responsible for lipidation-dependent degradation of APOB in hepatocytes. Our results support the use of these cell lines for studying cellular APOB and VLDL metabolism at an unprecedented resolution in hepatic cells.

## 2. Methods

### 2.1 Chemicals

Poly-L-lysine hydrobromide (PLL), glycine, bovine serum albumin (BSA), β-mercaptoethanol, tunicamycin, oleic acid-albumin, and polyethylenimine were purchased from Sigma-Aldrich (the Netherlands) (; paraformaldehyde (PFA) from Boster (the Netherlands); CP-346086 (CP) from Axon Medchem (the Netherlands); MG132 (MG) from Calbiochem (the Netherlands); and agarose was obtained from Invitrogen (the Netherlands). All chemicals are listed in Supplementary material online, *Table S1*.

### 2.2 Cell culture

HepG2, Huh-7, and HEK293T cells were obtained from ATCC (France) and cultured in Dulbecco's modified Eagle's medium (DMEM) supplemented with 10% foetal bovine serum and 10 000 U/mL penicillin-streptomycin (Gibco, the Netherlands). All cells were grown in a humidified environment at 37°C and 5% CO₂. To evaluate intracellular regulation and subsequent secretion of APOB, the cells were treated as indicated for 24 h, unless stated otherwise. These treatments consisted of 1 µM CP (an inhibitor of MTP), 5 µg/mL tunicamycin, 25 µg/mL MG, 0.4 mM oleic acid-albumin, or a combination of the aforementioned compounds. Vehicle control was used in all experiments. To measure cell proliferation, parental HepG2 and HepG2-APOB^mNeon^ cells were seeded in 6-well plates, and growth, assessed by confluence, was measured continuously for up to 96 h using an Incucyte S3 Live Cell Analyser (Sartorius, Germany).

### 2.3 Generation of hepatic cells that express endogenous APOB-mNeon

**Figure 1:**
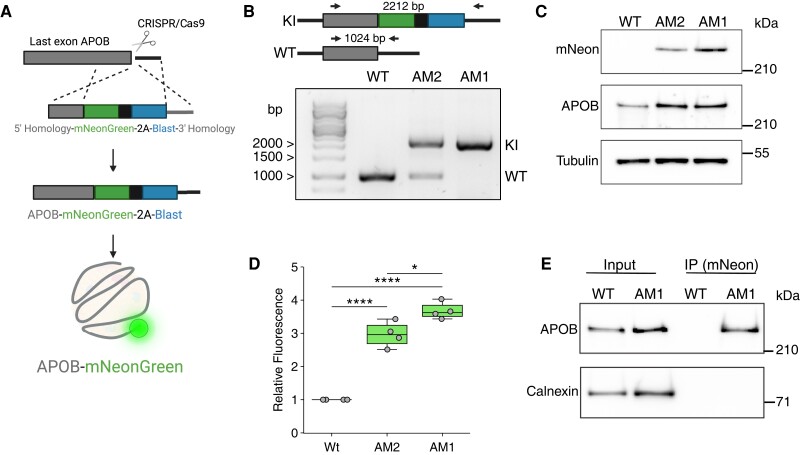
Generation and characterization of APOB-mNeon-producing hepatocytes. (*A*) A scheme depicting the engineering of hepatocytes to produce APOB-mNeon. A cassette containing mNeonGreen-P2A-Blast is integrated into the last exon of APOB using CRISPR/Cas9-mediated HDR. Correct integration of results in the production of APOB-mNeon protein. (*B*) Genomic DNA from clones with mono- and bi-allelic engineered APOB alleles is isolated and subjected to PCR (Clones AM2 and AM1, respectively). Amplification of the parental wild-type (WT) and engineered alleles results in a 1024 and 2212 bp amplicon, respectively. HepG2-APOB^mNeon^ clones are analysed by (*C*) immunoblotting of total cell lysates as indicated or (*D*) FACS analysis of cell-associated fluorescence (*n* = 4). (*E*) Total cell lysates from parental HepG2 (WT) and HepG2-APOB^mNeon^ clone AM1 are immunoprecipitated using an anti-mNeon antibody and immunoblotted as indicated. All immunoblots are representative of at least three independent experiments. (*D*) The box plots show the mean (middle line), 25th, 75th percentiles (box), and minimum and maximum values (whiskers). Data are analysed using a one-way ANOVA with a Tukey's test for multiple comparisons. *P < 0.05, ****P < 0.0001.

To engineer hepatocytes that express *APOB-mNeon*, we used a CRISPR-mediated homology-directed repair (HDR)-based strategy (*Figure 1A*). Briefly, we used SapI-based golden gate cloning to incorporate *APOB* homology arms into the donor plasmid TVBB C-term-mNeonGreen-P2A-Blast (see Supplementary material online, *Table S2*; Addgene #169229), as recently reported by Bollen *et al*.^19^ For each arm, an ∼500 bp homology sequence flanking the final coding exon of *APOB* was used. Integration of the homology arms resulted in the generation of TVBB-APOB_LHA_-mNeonGreen-P2A-Blast-APOB_RHA_. To induce a double-strand break in the last coding exon of *APOB*, an sgRNA was cloned into the Cas9-containing plasmid px330 (Addgene #42230; forward: 5′-CACCGCCTACATGAAGCTTGCTCCA-3′, reverse: 5′-AAACTGGAGCAAGCTTCATGTAGGC-3′). The correctness of all plasmids was confirmed by sequencing. The resulting px330-*APOB*_LE_ and TVBB-APOB_LHA_-mNeonGreen-P2A-Blast-APOB_RHA_ were transfected into hepatic cells at a 1:1 ratio. Cells were selected with 6 µg/mL blasticidin, and individual clones were isolated. To ensure correct integration of the donor template, we isolated genomic DNA from the individual clones using the QuickDNA microprep kit (Zymo Research, the Netherlands) and analysed it with polymerase chain reaction (PCR). A PCR using oligonucleotides external to the homology arms was designed so as to amplify the complete donor template (forward: 5′-CCATTCAGTCTCTCAAGACCAC-3′; reverse: 5′-ACCAGGGCTCGGAAGGTCTCTG-3′). This resulted in a 1024 bp amplicon in a non-edited allele and a 2212 bp fragment in a correctly edited allele. Additional PCRs were used to ensure correct integration into the *APOB* locus (not shown). Amplified fragments were isolated and sequenced to further ensure correct integration.

### 2.4 Immunoblotting analysis

Total cell lysates were prepared in a Radioimmunoprecipitation assay (RIPA) buffer (Boston BioProducts, Milford, MA)) supplemented with protease inhibitor cocktail (Roche, Switzerland) and 10 mM phenylmethylsulfonyl fluoride (Sigma, the Netherlands). The samples were cleared for 10 min at 4°C at 10 000 *g*. The cleared lysates were mixed (1:4 ratio) with a sample buffer (Invitrogen, the Netherlands) supplemented with 10% β-mercaptoethanol and subsequently warmed at 37°C for 10 min. Samples containing an equal amount of protein were loaded on NuPAGE 3–8% tris acetate gels (Invitrogen, the Netherlands) and transferred to nitrocellulose membranes. The membranes were blocked in TBST (10 mM Tris pH 8, 0.15 mM NaCl, 0.1% Tween-20) containing 5% milk and incubated with the primary antibodies in the same buffer for 16 h. Subsequently, the membranes were washed in TBST and incubated with horseradish peroxidase–conjugated secondary antibodies for 2 h. Blots were washed, visualized with chemiluminescence, and imaged on an Amersham ImageQuant800 (GE Healthcare, the Netherlands). The results shown are representatives of at least three independent repetitions, and protein bands were quantified using ImageJ software. All antibodies used in this study are listed in Supplementary material online, *Table S3*.

### 2.5 Immunoprecipitation analysis

Total cell lysates were prepared in a RIPA buffer as described above. To immunoprecipitate APOB-mNeon, we used mNeonGreen-Trap magnetic agarose beads following the manufacturer's guidelines (Chromotek, cat# ntma-20, the Netherlands). Briefly, the beads were washed once in a dilution buffer [10 mM Tris/Cl pH 7.5, 150 mM NaCl, 0.5 mM ethylenediaminetetraacetic acid (EDTA)], mixed with the cell lysate, and incubated for 16 h at 4°C while rotating. Subsequently, the beads were washed thrice with a wash buffer (dilution buffer supplemented with 0.05% NP40), and immunoprecipitated proteins were eluted by boiling beads at 95°C for 10 min in an SDS sample buffer. The eluted proteins were analysed by immunoblotting as described above.

### 2.6 Analysis of APOB-mNeon-containing VLDL secretion

To measure the secretion of fluorescent APOB-mNeon-containing lipoproteins, culture media were removed and replaced with FluoroBrite DMEM (Gibco). Medium was collected at the indicated time points and cleared by centrifugation at 4°C for 5 min at 10 000 *g*. Fluorescence intensity in the cleared medium was measured using a CLARIOstar plate reader (BMG LABTECH, Germany) with excitation set at 492 nm and emission detection at 537 nm. Fluorescence in culture media was subtracted to account for background fluorescence signal.

### 2.7 Flow cytometry analysis

To analyse intracellular APOB-mNeon-associated fluorescence, cells were washed twice in phosphate-buffered saline (PBS), detached using TripLE express (Gibco, the Netherlands), and resuspended in a fluorescence-activated cell sorting (FACS) buffer (PBS supplemented with 1% BSA and 5 mM EDTA). Subsequently, the cells were collected and fixed with 4% PFA for 10 min. The fixed cells were washed, resuspended in the FACS buffer, and measured on a CytoFLEX S Flow Cytometer (Beckman Coulter, the Netherlands) using the 488 nm laser for excitation and the 525 nm fluorescent channel for emission. Acquired data were analysed using FlowJo v10.8 Software (BD Life Sciences, the Netherlands). Single live cells for analysis were gated using standard forward scatter vs. side scatter gating.

### 2.8 Immunofluorescence analysis and imaging

For immunofluorescence imaging, cells were cultured on PLL-coated coverslips to 50% confluency. Subsequently, the cells were washed twice with PBS and fixed with 4% PFA in PBS for 15 min at room temperature. The fixed cells were washed in PBS, quenched with 0.15% glycine in PBS, and washed in PBS. Samples were then permeabilized with 0.05% triton in PBS for 10 min, blocked with 2% BSA for 10 min, and incubated with a primary antibody in 2% BSA for 1 h. Afterward, the samples were washed with PBS and incubated with a secondary antibody in 2% BSA for 1 h. Finally, the samples were washed in PBS, once with water, and mounted using a ProLong Glass Antifade Mountant with NucBlue Stain (Thermo Fisher, the Netherlands). The antibodies used are listed in Supplementary material online, *Table S3*. Images were taken using a Thunder Wide Field Fluorescent Imager (Leica) and analysed with ImageJ software.

### 2.9 Electron microscopy imaging of APOB

The procedure for cryo-sectioning and immunolabelling of target proteins was previously described.^20^ Briefly, cells were fixed by adding to a culture medium an equal volume of freshly 4% PFA in a 0.1 M phosphate buffer (pH 7.4; 2X solution). After 10 min, the fixative was replaced with 2% PFA in the 0.1 M phosphate buffer (pH 7.4; 1X solution) for 2 h at room temperature, after which samples were stored in 1% PFA at 4°C until further processing. After quenching with PBS/0.05 M glycine, the cells were scraped from the dish in 1% gelatin in PBS and pelleted in 12% gelatin in PBS. Cell pellets were then solidified on ice and cut into 1 mm^3^ blocks that were infiltrated overnight in 2.3 M sucrose for cryoprotection. Blocks were mounted on aluminium pins and frozen in liquid nitrogen. Ultrathin cryosections (80 nm) were made using Leica EM UC7 ultra cryotome, transferred on transmission electron microscopy grids with a 1:1 mixture of 2.3 M sucrose and 1.8% methylcellulose, and immunolabelled using a primary goat α APOB polyclonal antibody (Calbiochem #178467, 1:3000), followed by a secondary Rabbit anti-Goat IgG antibody (Nordic #RAG/7S, 1:1500). The secondary antibody was detected by Protein A conjugated to 15 nm gold particles (Cell Microscopy Core, Utrecht, The Netherlands). Pictures were collected on a JEM1010 (JEOL) equipped with a Veleta 2k × 2k CCD camera (EMSIS, Munster, Germany).

### 2.10 Endocytosis of APOB-mNeon-containing lipoproteins

An endocytosis of APOB-mNeon-containing lipoproteins into cells was conducted as previously reported by us.^21,22^ Briefly, HepG2-APOB^mNeon^ cells were grown in DMEM supplemented with lipoprotein-deficient serum (LPDS) for up to 7 days. Conditioned medium, enriched with APOB-mNeon-containing lipoproteins, was collected, filtered through a 45 µm mesh, and used immediately. To measure uptake, parental HepG2 cells were cultured for 16 h in DMEM supplemented with LPDS, 2.5 µg/mL simvastatin, and 100 µM mevalonate to enhance LDLR expression. Subsequently, the culture medium was replaced with conditioned medium collected from HepG2-APOB^mNeon^ cells. At the indicated time, the conditioned medium was removed, the cells were washed thrice in cold PBS, and they were either lysed with RIPA for immunoblotting, prepared for flow cytometry, or stained for immunofluorescence, as described in detail above.

### 2.11 Construction of ERAD-associated E3 ligase sgRNA library and E3 ligase screen

We used Chopchop (https://chopchop.cbu.uib.no) and MIT (https://www.zlab.bio) to design sgRNAs targeting 25 E3 ubiquitin ligases implicated in ERAD.^23^ For each ligase, three independent guides were designed. Additionally, two guides targeting *APOB* and *MTTP* were designed using Benchling (https://www.benchling.com). As control, a guide targeting the *AAVS* ‘safe harbour’ locus was used. The sequence of all sgRNAs used is listed in Supplementary material online, *Table S4*. The sgRNAs were cloned into pLentiCRISPRv2 (Addgene #52961), and the correctness of all constructs was verified by Sanger sequencing. Lentiviral particles were generated by HEK293T cells by co-transfecting the pLentiV2-sgRNA plasmids with 3rd generation packaging plasmids using polyethylenimine. After 16 h, the medium was replaced with DMEM containing 20 mM HEPES, and the medium containing lentivirus particles was collected and filtered through a 45 µm mesh. The lentivirus-containing medium was mixed 5:1 with DMEM and 12 μg/mL polybrene (Santa Cruz, the Netherlands) and used to transduce HepG2-APOB^mNeon^ cells (3× sgRNAs/gene pooled). Subsequently, the cells were selected with 3 μg/mL puromycin and used for the E3 ligase screen 5 days after the start of selection. To promote ERAD-dependent degradation of APOB, the cells were treated for 16 h with 1 µM CP before analysis by flow cytometry, as described above. To ablate *APOB* or *MTTP*, the same strategy was used, except that the cells were maintained under puromycin selection for 2 weeks.

### 2.12 Statistics

Statistical analysis was performed using Prism v10 (GraphPad, Boston, MA). Normal distribution of the data was tested using the Shapiro–Wilk test. The significance of the normally distributed data was analysed by one-way analysis of variance (ANOVA) in combination with a Tukey's multiple comparison test or a two-sided Student's *t*-test. Data were considered significant when *P* < 0.05. Significance is indicated with *P < 0.05, **P < 0.01, ***P < 0.001, and ****P < 0.0001.

## 3. Results

### 3.1 Generation and characterization of APOB-mNeon-producing cells

In an attempt to improve methods to study cellular APOB/VLDL metabolism, we opted to develop a set of hepatic cell lines in which endogenous APOB is fluorescently tagged. We used CRISPR/Cas9-mediated genome engineering to integrate an in-frame mNeon-encoding sequence into the last coding exon of *APOB* in hepatocyte-like HepG2 and Huh-7 cell lines (schematically illustrated in *Figure 1A*). Clonal selection resulted in the isolation of in-frame integrated mNeon in both hepatic cell lines, as confirmed by Sanger sequencing of PCR amplicons covering the mNeon integration site. All clones isolated from Huh-7 cells had retained a wild-type allele, whereas in HepG2 cells, mono- and bi-allelic edited clones were isolated (*Figure 1B* and Supplementary material online, *Figure S1A*). For this reason, subsequent experiments were conducted in HepG2-APOB^mNeon^ cells, and the results of key experiments were validated in Huh-7-APOB^mNeon^ cells. The proliferation of the genome-edited clones was indistinguishable from that of controls (see Supplementary material online, *Figure S2*). These cells produced APOB-mNeon protein that was absent in parental HepG2 cells but could be detected by immunoblotting with an anti-mNeon antibody or by FACS analysis in HepG2-APOB^mNeon^ cells (*Figure 1C* and *D*). We point out that the wild-type parental HepG2 cells shown in *Figure 1C* and *D* are not genome-edited and have not undergone clonal selection and only serve to demonstrate the specificity of APOB-mNeon detection. Furthermore, *APOB* mRNA levels were similar in clones AM1 (homozygous) and AM2 (heterozygous), indicating that the genome editing of *APOB* did not alter its expression (not shown). Consistent with clone AM2 retaining a wild-type *APOB* allele, the intensity of the APOB-mNeon signal was ∼50% of that detected in clone AM1, in which both alleles were edited. In line with the correct genome editing of Huh-7 clones, APOB-mNeon was detected in these cells by immunoblotting and FACS analysis (see Supplementary material online, *Figure S1B* and *C*). Finally, to confirm that the identified anti-mNeon signal indeed represents APOB, we enriched for mNeon with immunoprecipitation and followed this by immunoblotting for APOB. With this procedure, we detected a strong immunoprecipitated band in HepG2-APOB^mNeon^ cells. In contrast, this band was absent in parental HepG2 cells (*Figure 1E*).

**Figure 2:**
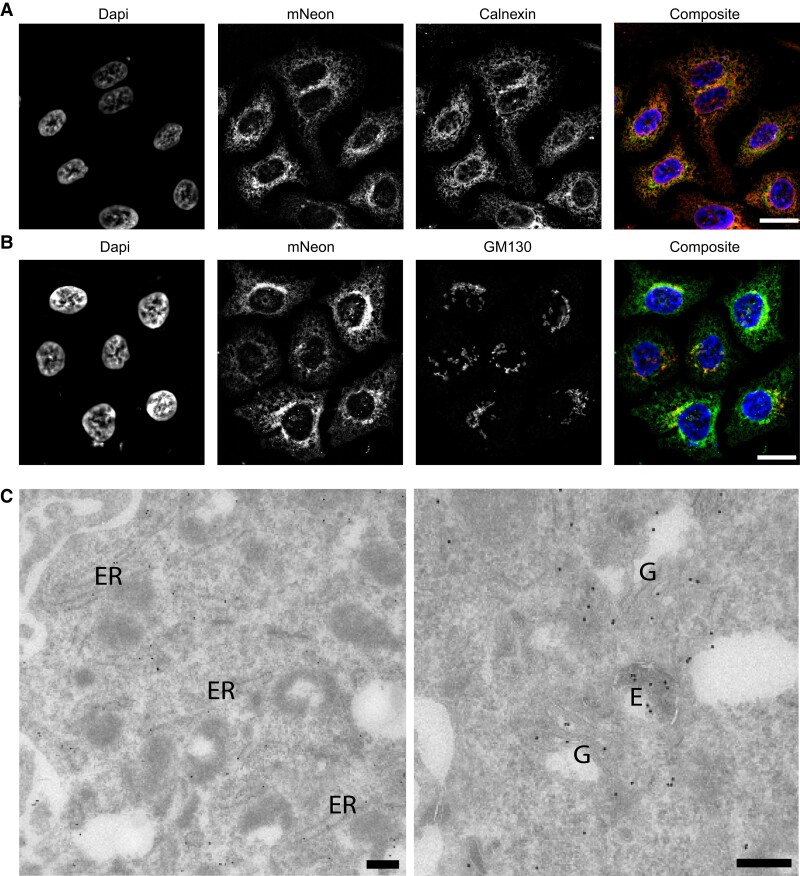
Intracellular localization of APOB-mNeon. (*A* and *B*) Cellular localization of APOB-mNeon was determined by immunofluorescence. Nuclei were counter-stained with Dapi (blue), APOB-mNeon (green), and (*A*) the ER marker Calnexin (red), or (*B*) the Golgi marker GM130 (red). Bar: 20 µm. (*C*) Immuno-EM. Ultrathin cryosections were immuno-gold labelled using an anti-APOB antibody (10 nm gold particles). APOB was found in the ER and Golgi complex (G), as well as in multi-vesicular endosomes (E). Bars, 100 nm.

Next, we aimed to determine the cellular localization of endogenous APOB-mNeon protein in HepG2-APOB^mNeon^ cells using immunofluorescence. The green signal emanating from APOB-mNeon could be easily visualized in both fixed and live cells. The APOB-mNeon signal co-localized predominantly with the ER marker Calnexin (*Figure 2A*) and to a lesser extent with the Golgi marker GM130 (*Figure 2B*). This suggests that the bulk of intracellular APOB is found in the ER. There is a paucity on the localization of endogenous APOB in human cells and in hepatocytes in particular. Therefore, to further study the localization of APOB-mNeon in ultrastructural detail, we performed immuno-electron microscopy (immuno-EM) using immuno-gold labelling of ultrathin cryosections. Unfortunately, the anti-mNeon antibody was not compatible with immuno-EM, which is why we used an anti-APOB antibody instead. As all APOB alleles have been edited in the HepG2-APOB^mNeon^ clone AM1 (*Figure 1B*), this reflects the localization of APOB-mNeon in the engineered cells. We immuno-detected APOB-mNeon predominantly in the ER and Golgi stacks (*Figure 2C*). Furthermore, APOB was found at the plasma membrane and occasionally in clathrin-coated pits (see Supplementary material online, *Figure S3A*). In addition, APOB was found in multi-vesicular endosomes, likely reflecting endocytosed APOB (*Figure 2C* and Supplementary material online, *Figure S3B*). Collectively, these results confirm that we generated APOB-mNeon-producing hepatic cells, which can be used for studying endogenous APOB intracellularly.

### 3.2 Intact (post)transcriptional regulation of APOB-mNeon

**Figure 3:**
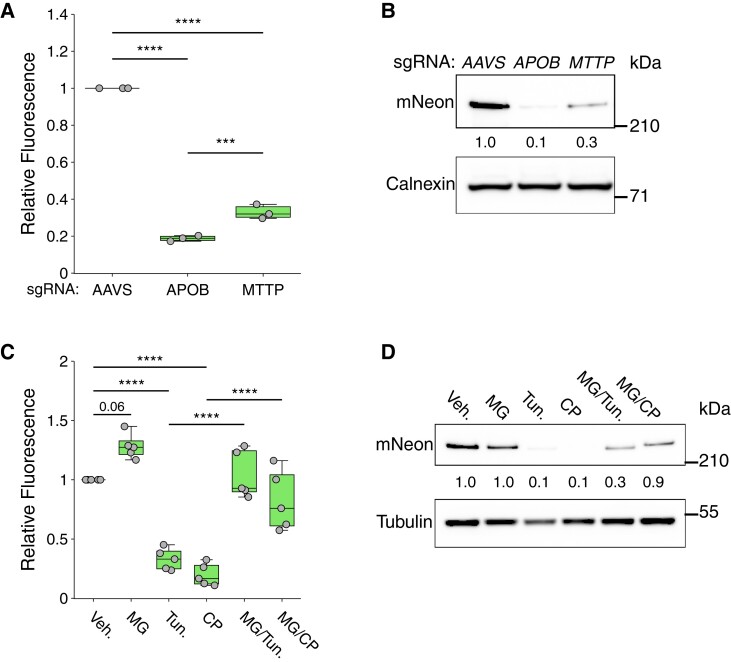
Intact (post)transcriptional regulation of APOB-mNeon. (*A* and *B*) Analysis of intracellular APOB-mNeon level in HepG2-APOB^mNeon^ cells following ablation of *APOB*, *MTTP*, or *AAVS* as control by (*A*) FACS or (*B*) immunoblotting of total cell lysates as indicated. (*C* and *D*) HepG2-APOB^mNeon^ cells were treated for 24 h with vehicle (Veh.), 5 µg/mL tunicamycin (Tun.), and 1 µM CP in the presence or absence of 25 µg/mL MG added during the last 6 h. The level of intracellular APOB-mNeon was determined by (*C*) FACS, or by (*D*) immunoblotting of total cell lysates as indicated. (*B* and *D*) All immunoblots are representative of at least three independent experiments, with the mean intensity of APOB-mNeon relative to vehicle control indicated. (*A* and *C*) The box plots show the mean (middle line), 25th, 75th percentiles (box), and minimum and maximum values (whiskers). The fluorescent intensity of control was set to 1. Data were analysed using one-way ANOVA with a Tukey's test for multiple comparisons. ***P < 0.001, ****P < 0.0001.

Having generated a set of APOB-mNeon hepatic cell lines, we questioned whether these cells could be used to interrogate the (post)transcriptional regulation of endogenous APOB. For this, we evaluated known genetic, pharmacologic, and physiological regulators of APOB. The MTP-mediated co-translational lipidation of APOB is a central determinant of its stability, as diminished lipidation results in rapid APOB proteasomal degradation.^7,8^ Consistent with this, CRISPR-mediated deletion of *MTTP* in HepG2-APOB^mNeon^ cells led to a marked decrease in cellular APOB, as assessed by both FACS and immunoblotting (*Figure 3A* and *B*), in the absence of compensatory changes in SREBP signalling (not shown). This decrease was comparable to that observed when we ablated *APOB* itself. Next, we evaluated whether the regulation of APOB-mNeon in HepG2-APOB^mNeon^ cells in response to established perturbations is intact. Herein, we evaluated the response to pharmacological inhibition of MTP by the high-affinity inhibitor CP, the glycosylation inhibitor tunicamycin which induces ER stress (Tun), and proteasomal inhibition by MG.^24–26^ Inhibition of MTP activity, similar to its genetic ablation, and of glycosylation has been previously reported to promote the ubiquitination and subsequent proteasomal degradation of APOB.^24,26^ Accordingly, treatment with CP and tunicamycin dramatically decreased the abundance of detectable APOB-mNeon (*Figure 3C* and *D*). Inhibiting proteasomal activity alone with MG resulted in a trend towards increased APOB-mNeon protein (*P =* 0.06). The same treatment could largely reverse the decrease in APOB-mNeon levels in response to CP and tunicamycin, in line with these treatments promoting the ERAD-mediated degradation of APOB in HepG2-APOB^mNeon^ cells. In aggregate, these results indicate that the regulation of cellular APOB is intact in the developed cells, and that these can be readily used for interrogating APOB using genetic and pharmacologic perturbations.

### 3.3 Regulated secretion of functional APOB-mNeon-containing lipoprotein particles

**Figure 4:**
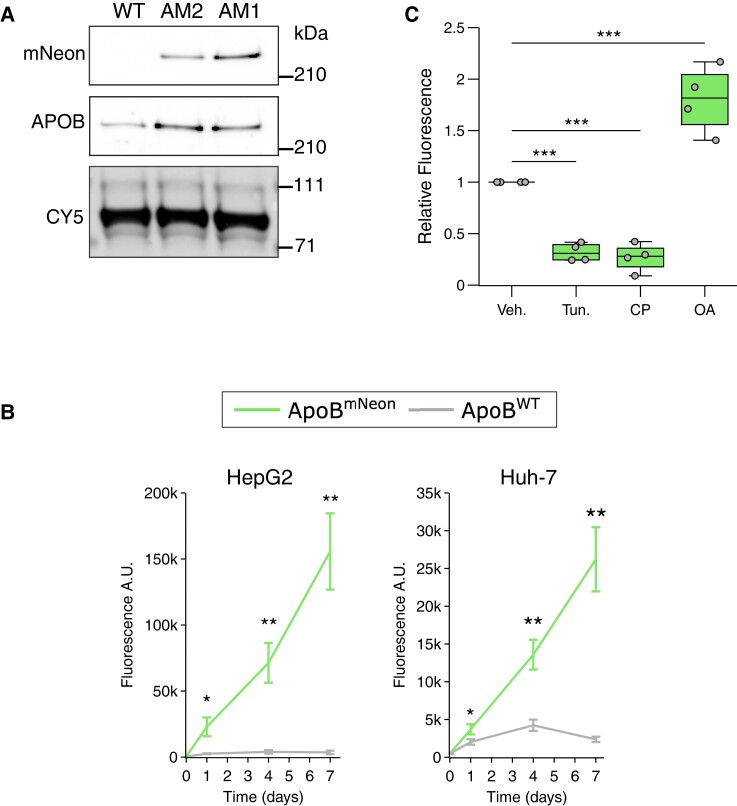
Regulated secretion of APOB-mNeon-containing lipoprotein particles. (*A*) Culture medium was collected from Clones AM1 and AM2 and immunoblotted as indicated. Total cell lysates were prepared from the same cells, and Cy5 labelling of proteins in culture media was used to control for equal loading. Immunoblots are representative of at least three independent experiments. (*B*) HepG2-APOB^mNeon^ and Huh7-APOB^mNeon^ clones, or their corresponding parental lines were cultured as shown. The culture media were sampled at the indicated time points, and the fluorescent signal determined. Each point and error represents the mean ± standard deviation (SD) of three independent experiments done in triplicate. (*C*) HepG2-APOB^mNeon^ cells were treated for 24 h with vehicle (Veh.), 5 µg/mL tunicamycin (Tun.), 1 µM CP, or 0.4 mM oleic acid. Subsequently, culture media were collected and the APOB-mNeon-derived fluorescence measured and plotted relative to vehicle control which was set at 1. The box plots show the mean (middle line), 25th, 75th percentiles (box), and minimum and maximum values (whiskers). Data were analysed using one-way ANOVA with a Tukey’s test for multiple comparisons. *P < 0.05, **P < 0.01, ***P < 0.001.

Having established that the developed cells can be used to study intracellular APOB, we tested whether these cells can also be used in parallel to study the secretion of APOB-containing lipoproteins. For this, we first collected culture media from HepG2-APOB^mNeon^ cells and used immunoblotting to test whether secreted APOB-mNeon could be detected. A comparable level of APOB was detected in media from the two independent HepG2-APOB^mNeon^ clones (*Figure 4A*). As anticipated, APOB-mNeon was only detected in culture media collected from APOB-mNeon-producing HepG2 and Huh-7 edited clones (*Figure 4A* and Supplementary material online, *Figure S1B*). Notably, the level of secreted APOB-mNeon was ∼50% lower in the heterozygous clone (clone AM2) then in the homozygous clone (AM1).

Next, we determined the time-dependent secretion profile of APOB-mNeon in the genome-edited HepG2 and Huh-7 cells. Secretion of APOB-mNeon into the culture medium increased linearly over time and could be followed for up to 7 days in HepG2 and Huh-7 cells (*Figure 4B*). We then questioned whether the treatments used to evaluate the intracellular levels of APOB-mNeon (*Figure 3*) are mirrored by a corresponding change in its secretion. Reduced intracellular APOB-mNeon following tunicamycin and CP treatment was directly coupled to a decrease in fluorescent lipoproteins in the culture medium (*Figure 4C*). Conversely, oleic acid treatment promoted the secretion of APOB-mNeon lipoproteins, as previously reported.^27,28^ Of note, we did not observe any overt change in intracellular localization of APOB-mNeon in response to exogenous oleic acid treatment (not shown). Collectively, these results demonstrate that the developed cells can be used for the parallel quantification of both intra- and extra-cellular APOB.

**Figure 5:**
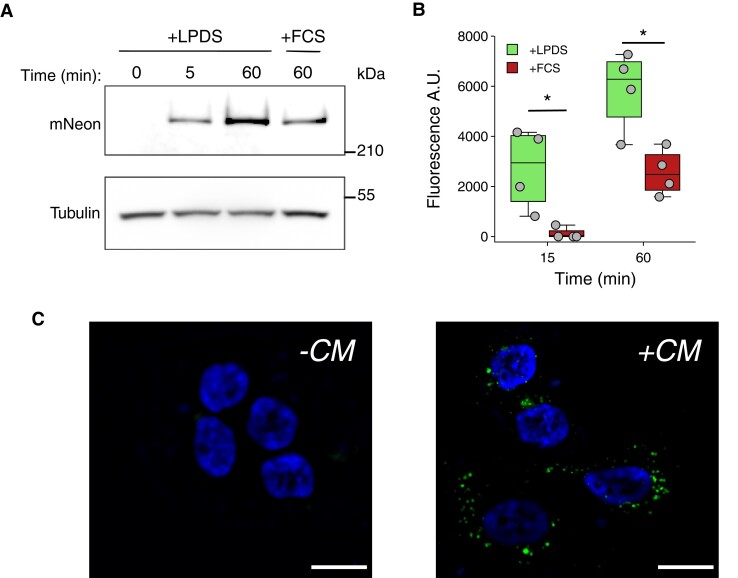
HepG2-APOB^mNeon^ cells produce APOB-mNeon-containing VLDL that can be endocytosed. (*A*) Conditioned culture media (CM) from HepG2-APOB^mNeon^ cells that were cultured with lipid-deficient medium for 6 days was collected and filtered. Subsequently, sterol-depleted parental HepG2 cells were cultured for the indicated time with CM supplemented with 10% LPDS or 10% FCS, extensively washed and total cell lysates immunoblotted as indicated (*n* = 3 independent experiments). A representative immunoblot is shown. (*B* and *C*) Parental HepG2 cells were cultured as described in (*A*), and the time-dependent uptake of ApoB-mNeon from CM was determined. Each point and error represents the mean ± SD (*n* = 4 independent experiments done in triplicate), or (*C*) imaged for cellular uptake of ApoB-mNeon-containing lipoprotein particles (±CM) as indicated. Data were analysed using Welch's *t*-test.*P < 0.05.

We then asked whether the secreted APOB-mNeon-containing particles produced by HepG2-APOB^mNeon^ cells could be endocytosed. For this, we cultured parental HepG2 cells in lipoprotein-depleted medium and determined their ability to take up fluorescent lipoproteins present in conditioned media collected from HepG2-APOB^mNeon^ cells. Already within 5 min, we were able to detect endocytosed APOB-mNeon protein in cell lysates of parental cells (*Figure 5A*). The signal further increased with a longer incubation time and could be competed off by the addition of excess lipoproteins in serum. The time-dependent endocytosis of APOB-mNeon-containing particles could also be quantified by FACS analysis, and evident by immunofluorescence imaging of cells (*Figure 5B* and *C*). Notably, with electron microscopy, we also observed the presence of APOB on the plasma membrane, within clathrin-coated pits, and inside multi-vesicular bodies (see Supplementary material online, *Figure S3A* and *B*). This localization is also consistent with endocytosis of ApoB-mNeon-containing lipoproteins by hepatocytes. Overall, these results indicate that HepG2-APOB^mNeon^ cells produce functional APOB-mNeon-containing APOB that can be endocytosed.

### 3.4 An ERAD-focused genetic screen to identify determinants of APOB degradation

**Figure 6:**
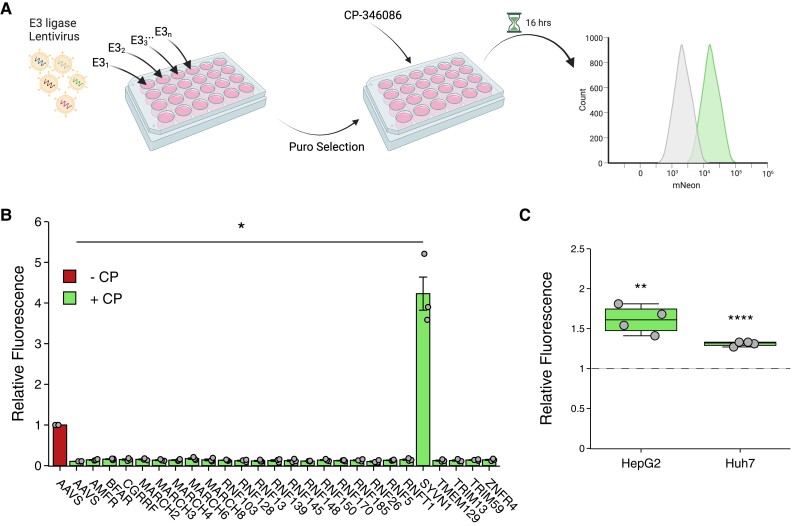
An ERAD-focused E3 ligase sgRNA screen identifies SYVN1 as a key determinant of lipidation-dependent APOB degradation. (*A*) A scheme depicting the key steps used for identifying E3(s) involved in lipidation-dependent degradation of APOB-mNeon. Briefly, HepG2-APOB^mNeon^ cells were plated and subsequently transduced with pooled lentiviral particles (3x sgRNAs/E3/well). The cells were selected with puromycin for 5 days and then treated with 1 µM CP for 16 h. The cells were then detached and intracellular APOB-mNeon fluorescence was measured using FACS. (*B*) The fluorescence signal in the presence of CP (+CP, second bar) relative to that in AAVS control without CP (−CP, first bar) was plotted. The mean ± SD of three independent experiments is shown. (*C*) The level of intracellular APOB-mNeon was determined by FACS in control *AAVS*- and *SYVN1*-ablated HepG2-APOB^mNeon^ and Huh7-APOB^mNeon^ cells cultured in the absence of CP. The abundance of APOB-mNeon in each cell line is plotted relative to the corresponding *AAVS* control (dashed line) which was set at 1. The box plots show the mean (middle line), 25th, 75th percentiles (box), and minimum and maximum values (whiskers). Data were analysed using (*B*) one-way ANOVA with a Tukey's test for multiple comparisons, or (*C*) two-sided Student's *t*-test vs. corresponding *AAVS* control.*P < 0.05, **P < 0.01, ****P < 0.0001.

Non-lipidated APOB is subject to rapid ubiquitylation in the ER and subsequent proteasomal degradation. However, the identity of the E3 ubiquitin ligase(s) responsible for this step is unclear. Therefore, as a proof of concept for the utility of the genome-engineered hepatocytes, we aimed to identify ERAD-associated E3 ubiquitin ligase(s) responsible for the lipidation-dependent degradation of APOB. To do so, we constructed a dedicated lentiviral sgRNA library targeting all 25 identified membrane-resident ER-resident E3 ligases (3 sgRNAs/E3).^23^ As control, we targeted the ‘safe-harbour’ *AAVS* locus. HepG2-APOB^mNeon^ cells were transduced with this library in an arrayed manner (i.e. 1xE3/well) and subsequently treated with CP to inhibit MTP-mediated lipidation of APOB-mNeon, as schematically shown in *Figure 6A*. As described above, the treatment with the MTP inhibitor reduces the level of intracellular APOB-mNeon due to enhanced proteasomal degradation (*Figure 6B*; compare AAVS −CP (first bar) to AAVS +CP (second bar). Of the 25 ligases evaluated, only ablation of *SYVN1* (also known as HRD1) prevented the MTP inhibitor-induced degradation of APOB. Deletion of *SYVN1* in the absence of CP treatment also slightly increased the basal level of APOB-mNeon in HepG2 and Huh7 genome-edited cells (*Figure 6C*). The higher levels of intracellular APOB-mNeon in HepG2 cells did not result in elevated secretion of APOB-mNeon (see Supplementary material online, *Figure S4*). Taken together, these results suggest that the E3 ligase SYVN1/HRD1 is part of a homeostatic mechanism that controls the physiological abundance of APOB in hepatocytes and highlights the potential of conducting genetic screens in the APOB-mNeon cell lines.

## 4. Discussion

In this study, we report the development of a suite of genetically engineered human hepatic cells to study the spatiotemporal metabolism of endogenous APOB/VLDL production and secretion. We demonstrate that endogenously produced APOB-mNeon retains its known physiological regulation and results in the secretion of lipoprotein particles that can be endocytosed. Showcasing the usefulness of these engineered cells, we conducted an ERAD-focused CRISPR screen that resulted in the implication of the E3 ubiquitin ligase SYVN1 in lipidation-dependent proteasomal degradation of APOB. Collectively, this highlights the utility of the developed engineered cells as valuable models for further research and exploration of hepatic APOB-related processes.

The lack of tractable hepatocyte-based models to study endogenous APOB has hampered studying its intracellular metabolism. Walsh *et al.*^17^ recently reported the generation of Cos-7 cells stably over-expressing APOB-GFP constructs that were used for studying both intracellular and secreted APOB. A limitation of this cell-based system is the use of an APOB over-expression approach, and the reliance on a cell type in which APOB is not physiologically expressed. Different from Cos-7 cells, HepG2 and HuH-7 cells are extensively used as human hepatocyte-like models and are also established for studying assembly and secretion of APOB-containing VLDL^27,29^ and of other genes implicated in the development of MALSD, like *PNPLA3* and *TM6SF2*.^30–32^ Hence, the development of genome-engineered hepatic APOB-mNeon cells, reported in this study, allows studying the transcriptional and post-transcriptional regulation of *APOB* in cells with hepatic machinery. As a tool, these cells facilitate the parallel interrogation of both intracellular and secreted APOB using common experimental strategies. An inherent limitation of using the immortalized human hepatic cell lines, or for that matter, any other non-physiological heterologous expression system, is the known production and secretion of lipid-poor VLDL particles.^27,28^ With the exception of VLDL-sized particles that are produced in rat McArdle cells,^33,34^ these particles do not fully mimic VLDL in humans. To a certain degree, this challenge can be mitigated by culturing the same hepatic cell lines used in our study with human serum, a condition that ‘humanizes’ their lipoprotein metabolism.^35,36^ Needless to say, any cell-based model will not be able to fully reproduce the *in vivo* setting and to replace the use of animal models, in which the intracellular and systemic metabolism of APOB/VLDL is intact. However, for hepatocyte-intrinsic factors that control assembly and secretion of APOB-containing VLDL, the engineered cells developed here represent an attractive experimental discovery and validation model.

The ubiquitin proteasomal system has been implicated in the regulated degradation of key nodes of lipid metabolism.^37^ Notable examples are the stimulated degradation of the LDLR by the E3 ubiquitin ligase Inducible degrader of the LDLR (IDOL),^38,39^ and of the rate-limiting enzymes in the mevalonate pathway HMGCR (by GP78, TRC8, HRD1, RNF145)^40^ and SQLE (MARCHF6).^41,42^ Similarly, the lipidation-dependent ERAD of APOB is a central determinant of VLDL assembly and subsequent secretion.^43^ In a seemingly futile cycle, APOB is continuously produced and unless adequately lipidated is ubiquitylated and degraded in cytosolic proteasomes after its extraction from the ER lumen. The E3 ligase GP78 has been proposed to mediate this process, as its over-expression in HepG2 cells stimulates the degradation of APOB.^44^ Reciprocally, silencing of *GP78* attenuated the appearance of poly-ubiquitylated APOB forms but did not increase abundance of APOB protein.^9^ Hence, despite ERAD of APOB being a key step in VLDL assembly, the conclusive identity of the E3 ligase(s) responsible for lipidation-dependent ubiquitylation of APOB is still elusive. As a showcase for the utility of the developed cells, we therefore questioned which of the 25 membrane-associated ERAD-implicated E3 ligases is responsible for this activity.^23^ Using an ERAD-focused CRISPR library, we found that SYVN1 (also known as HRD1) is the E3 responsible for the degradation of poorly lipidated APOB in HepG2-APOB^mNeon^ cells. In this setting of MTP inhibition, ablation of *GP78* had no effect on intracellular APOB-mNeon. SYVN1/HRD1 has, thus, far not been directly implicated in the ERAD of APOB. However, one earlier study reported that SYVN1 and APOB interact by immunoprecipitation in HepG2 cells, in line with APOB being a potential target for HRD-mediated ubiquitylation.^45^ In mice, global deletion of SYVN1 results in embryonic lethality.^46^ Liver-specific HRD1 knockout mice have been reported and display a complex metabolic phenotype, owing to the fact that HRD1 has multiple important and interlinked metabolic targets.^47^ Unfortunately, the level of APOB in the livers or plasma of these mice was not reported. In humans, no diseases or lipid-associated traits have been associated with mutations in SYVN1,^48^ even though levels of this E3 may be involved in the development of multiple sclerosis and Alzheimer's disease.^49,50^ Further studies on the role of SYVN1/HRD1 in VLDL assembly and secretion are therefore clearly warranted.

In conclusion, in this study, we report the development of a suite of genome-engineered hepatic lines that produce endogenous APOB-mNeon and report on its localization, levels, and secretion. These cells can be used to interrogate the assembly and secretion of APOB-containing particles in a quantitative manner and empower the discovery of new determinants and therapeutic targets in these processes that are amenable to pharmacologic and genetic perturbations.

**Translational perspective**

Dysregulated hepatic production of apolipoprotein B (APOB)-containing very LDL (VLDL) particles is a central determinant of plasma lipoprotein levels and development of atherosclerosis. The mechanisms underlying VLDL production are not fully understood, emphasizing the need for tractable human hepatocyte models to study this. In this study, we developed a suite of genome-engineered human hepatocytes that produce APOB-mNeonGreen, which allows the physiologic, genetic, and pharmacologic interrogation of APOB/VLDL metabolism. With these cells, we identified SYVN1 as the E3 ubiquitin ligase responsible for the degradation of lipid-poor APOB. Our study highlights the use of developed models for high-throughput discovery of genetic and pharmacologic interventions to target hepatic VLDL production.

## Supplementary Material

cvae121_Supplementary_Data

## Data Availability

All data supporting the findings of this study are available within the manuscript and its Supplementary Material.
