# Supplementary material for: A suite of genome-engineered hepatic cells provides novel insights into the spatiotemporal metabolism of apolipoprotein B and apolipoprotein B–containing lipoprotein secretion
Source: Cardiovasc Res. 2024 Jun 4;120(11):1253–64. doi: 10.1093/cvr/cvae121 (PMC11416059; doi:10.1093/cvr/cvae121)

Source image data

A suite of genome-engineered hepatic cells provide novel insights into the spatiotemporal metabolism of APOB and APOB-containing lipoprotein secretion

Meurs et al.

Figure 1. C

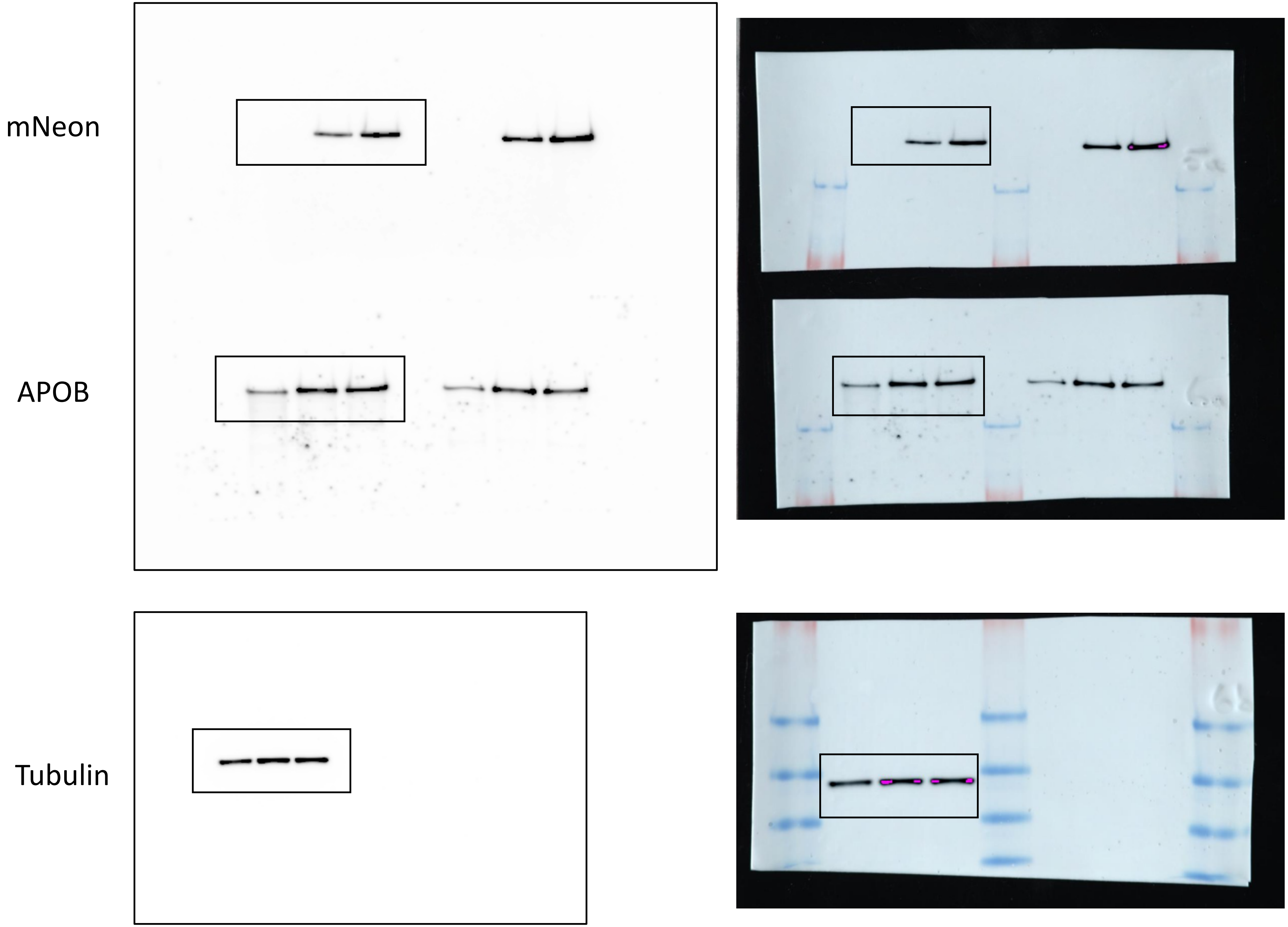

Figure 1. E

mNeon

Calnexin

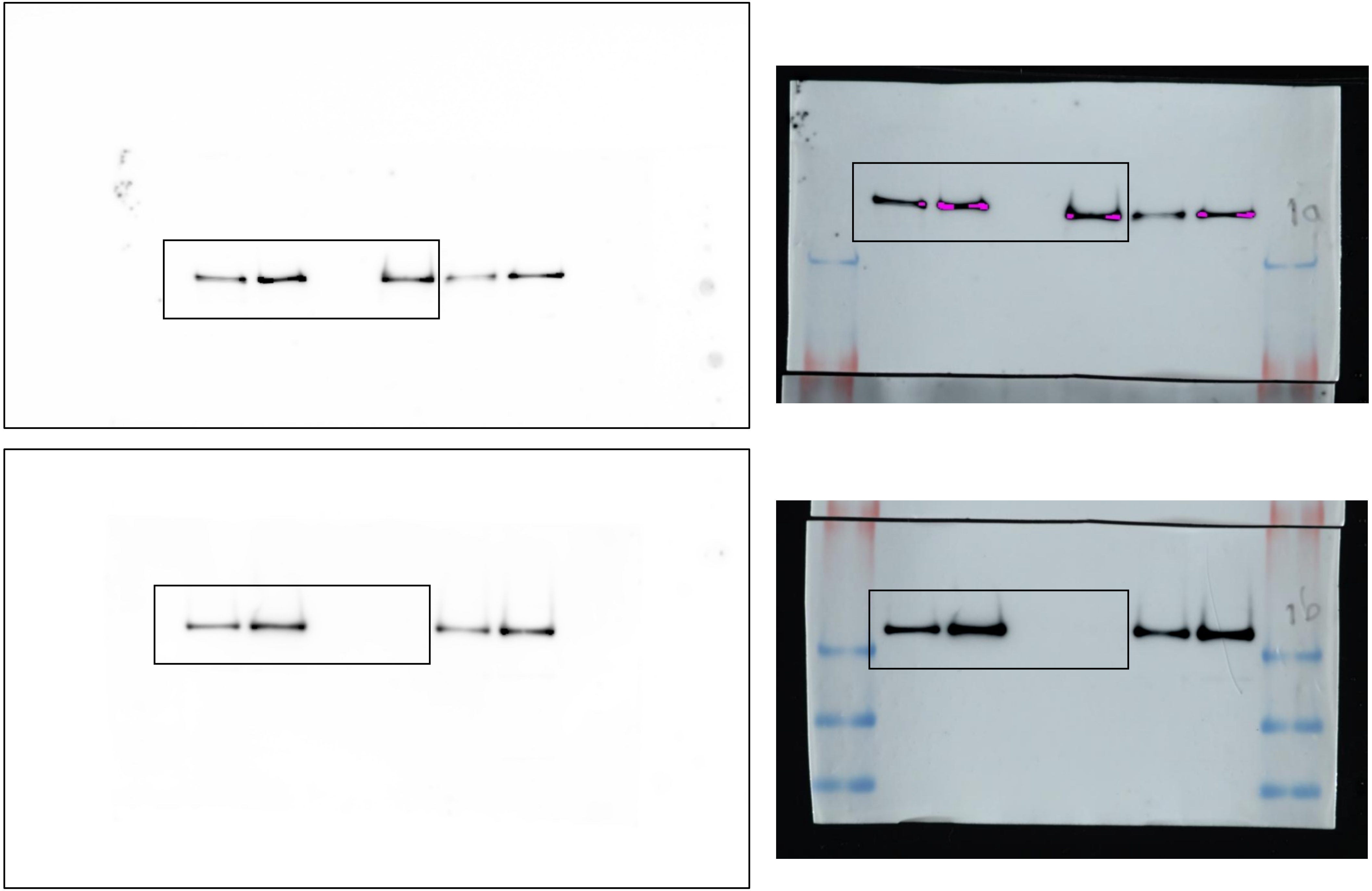

Figure 3. B

mNeon

Calnexin

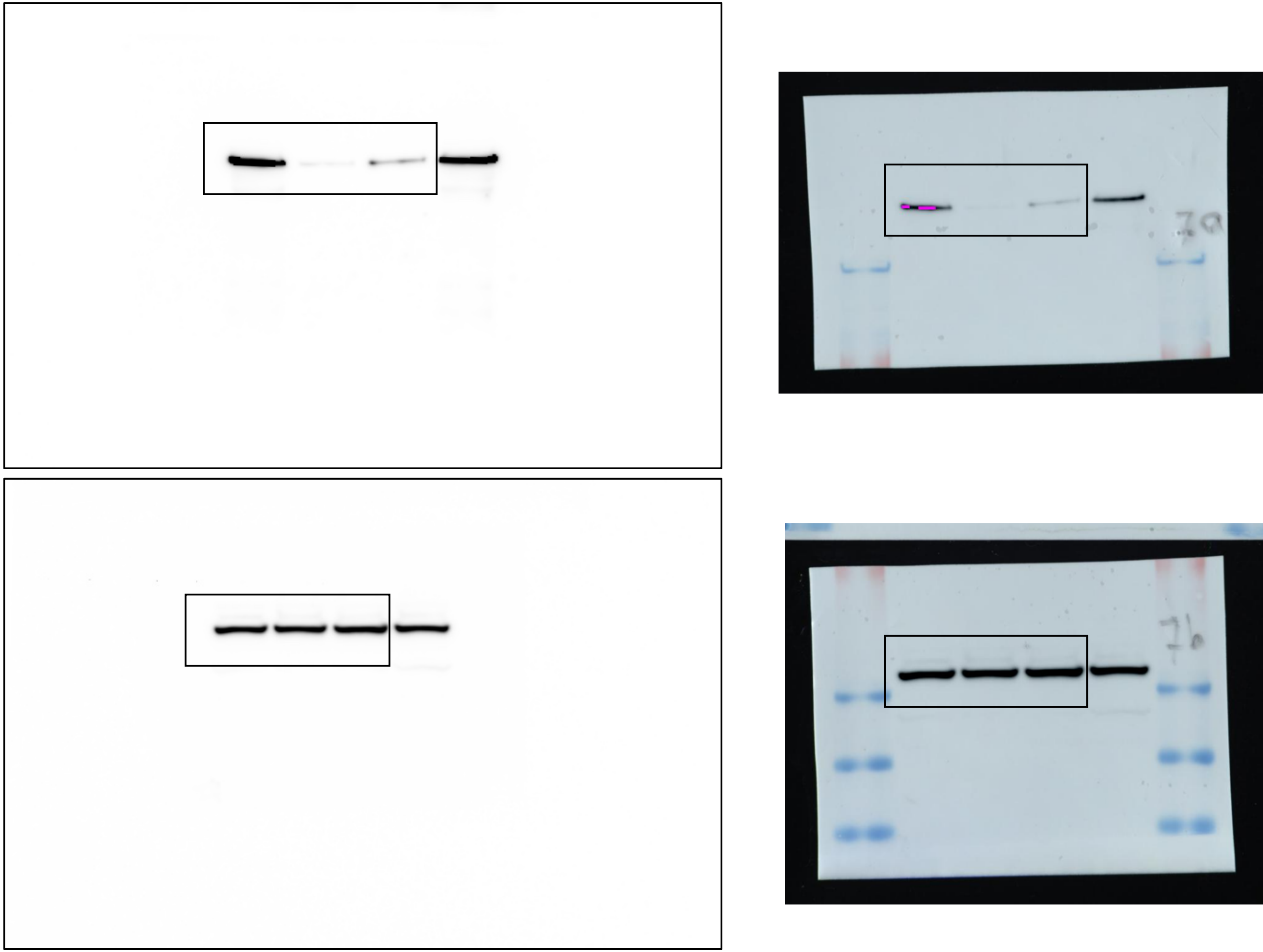

Figure 3. D

mNeon

Tubulin

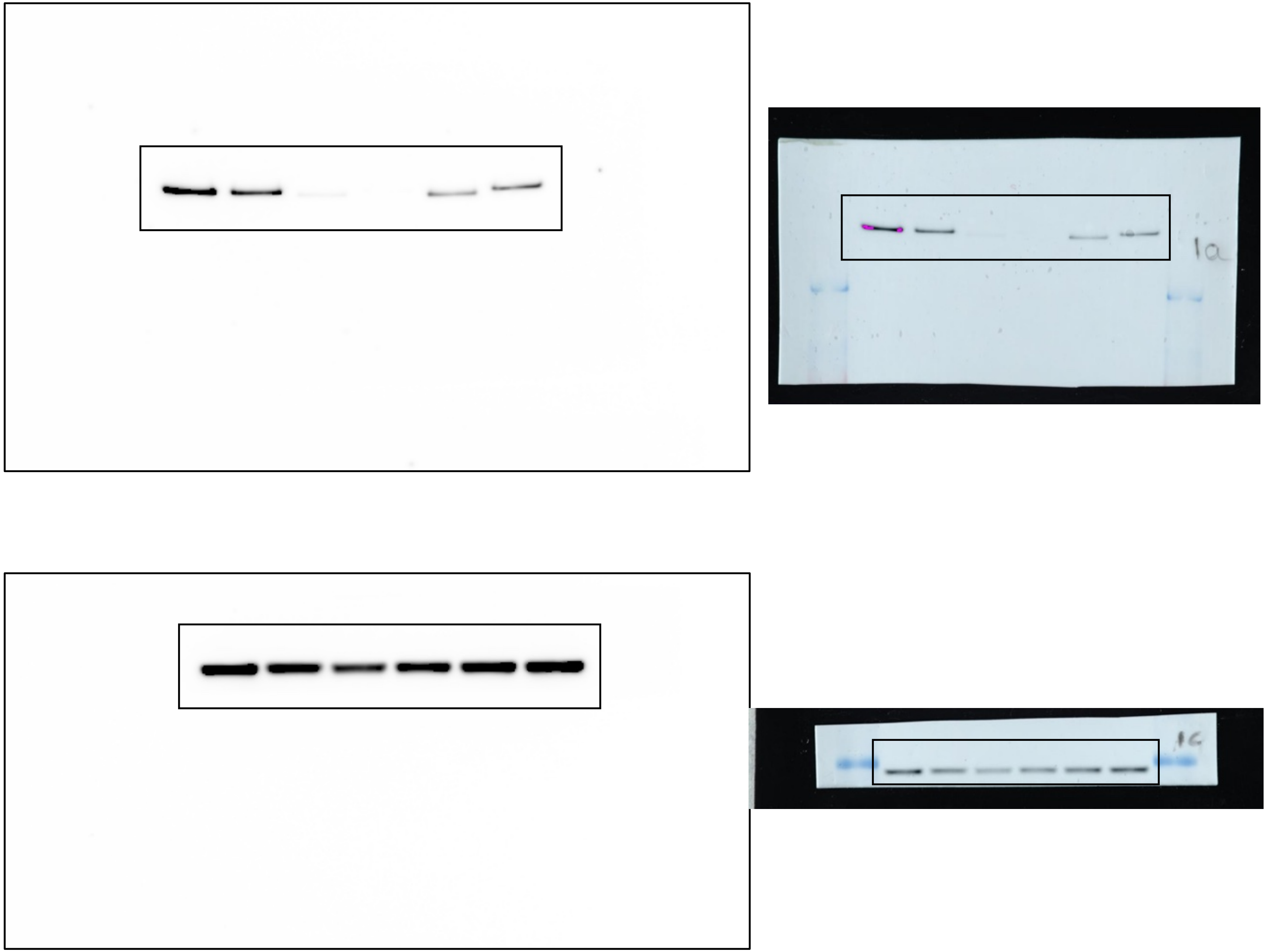

Figure 4. A

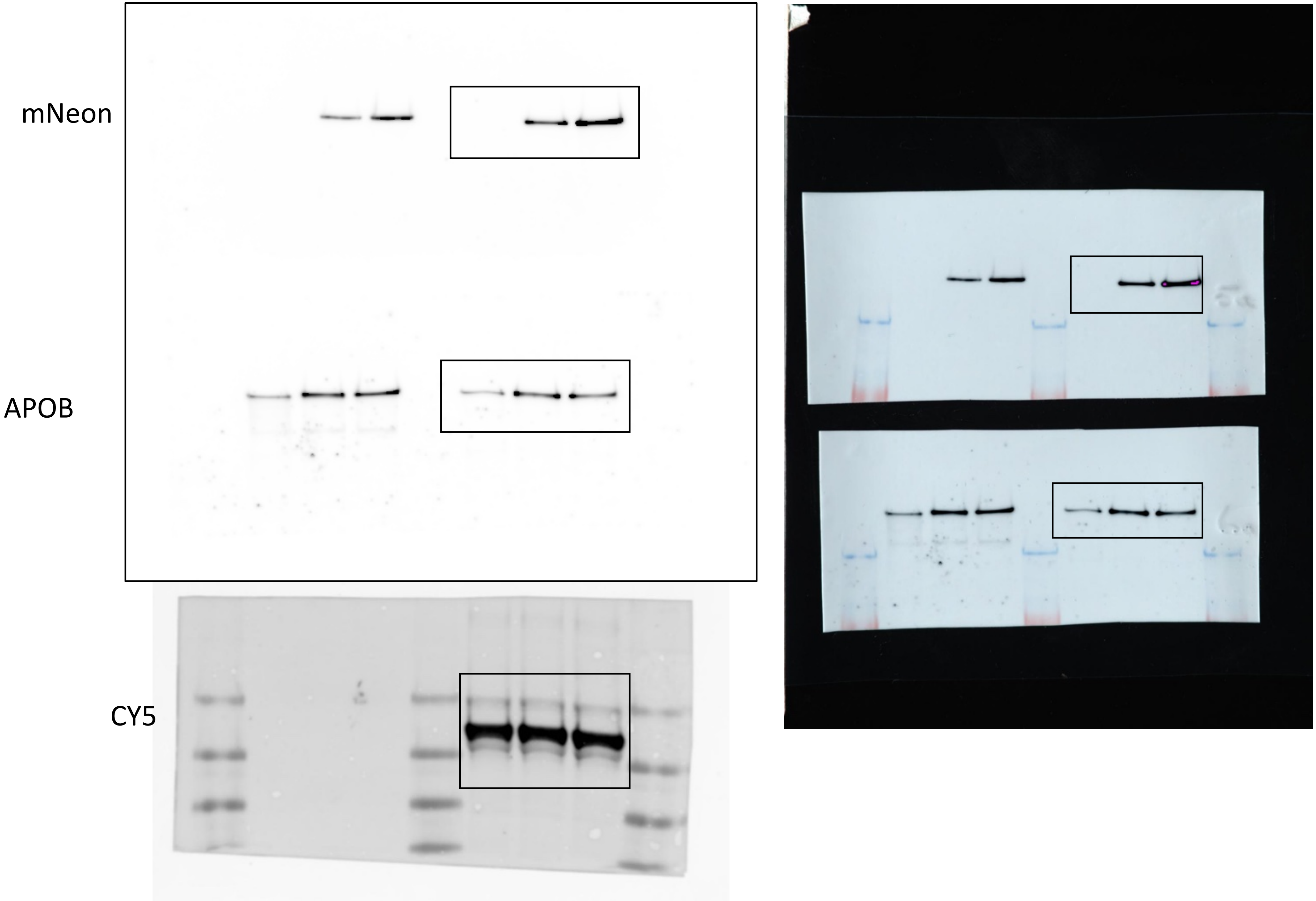

Figure 5. A

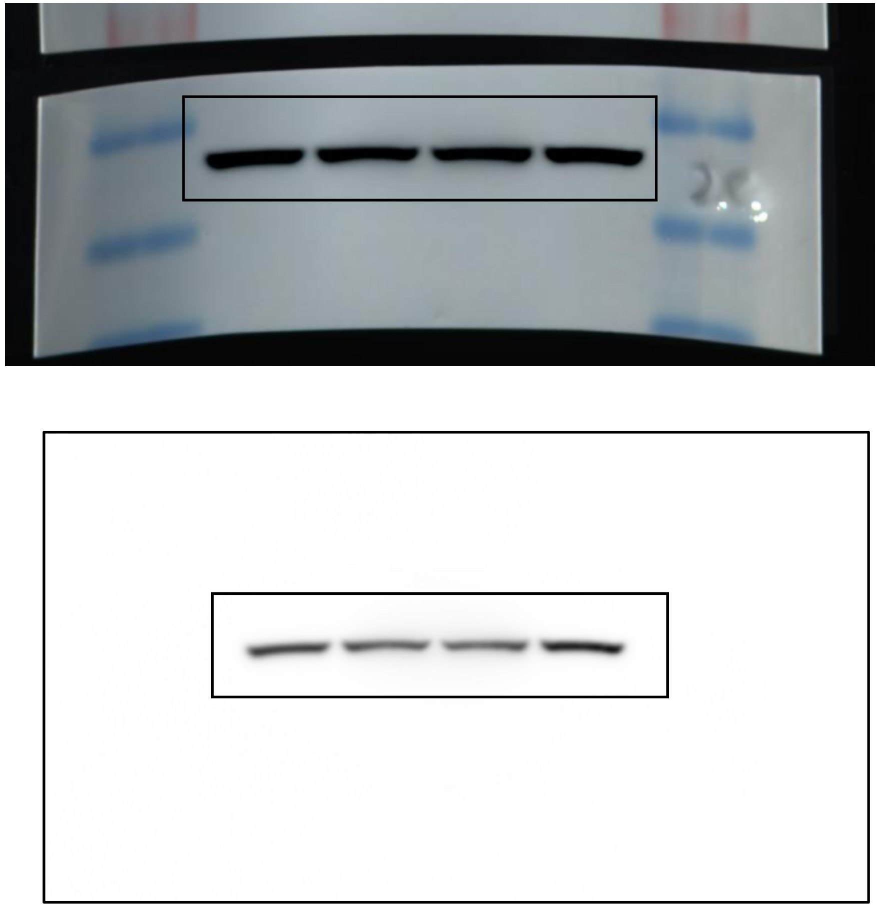

Tubulin

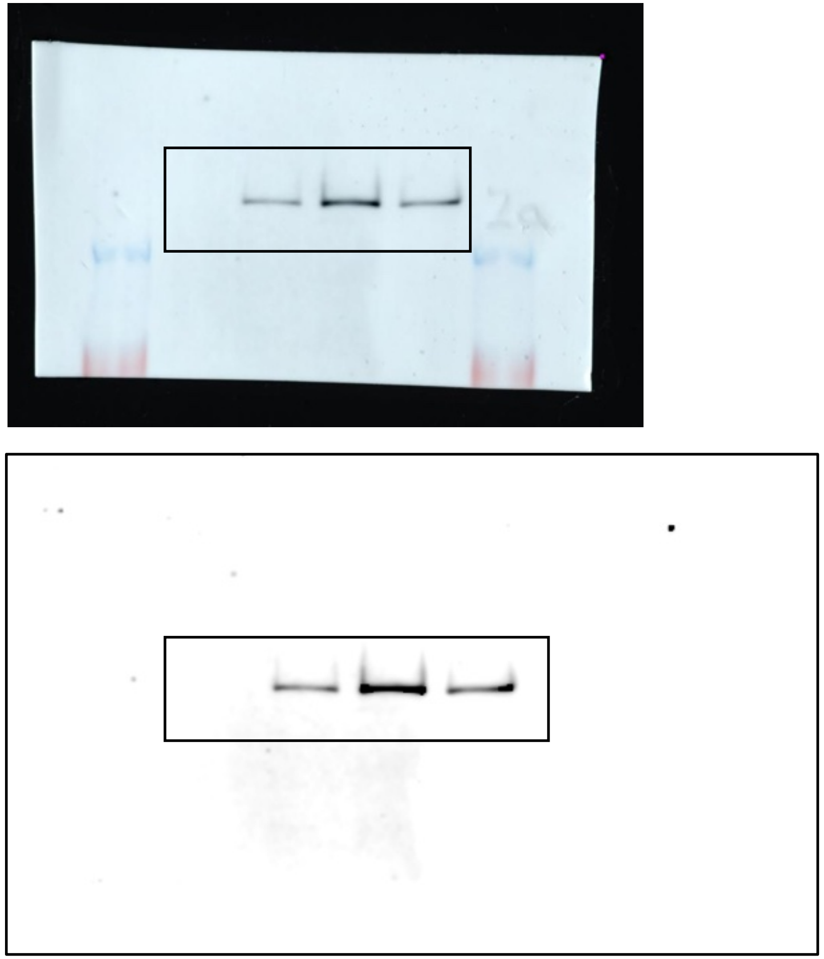

mNeon

Supplemental Figure 1. B

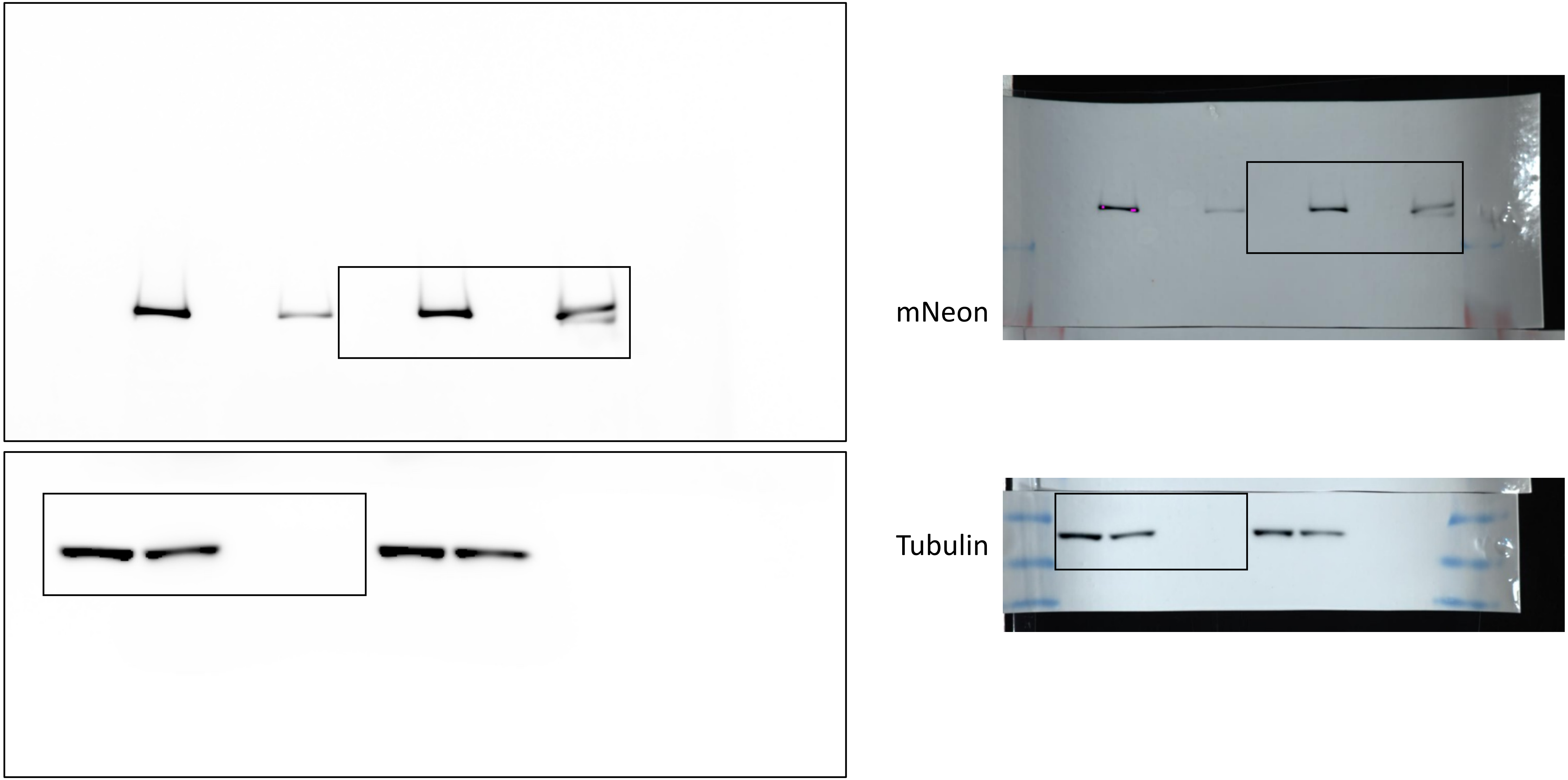

Supplemental Figure 1. D

mNeon

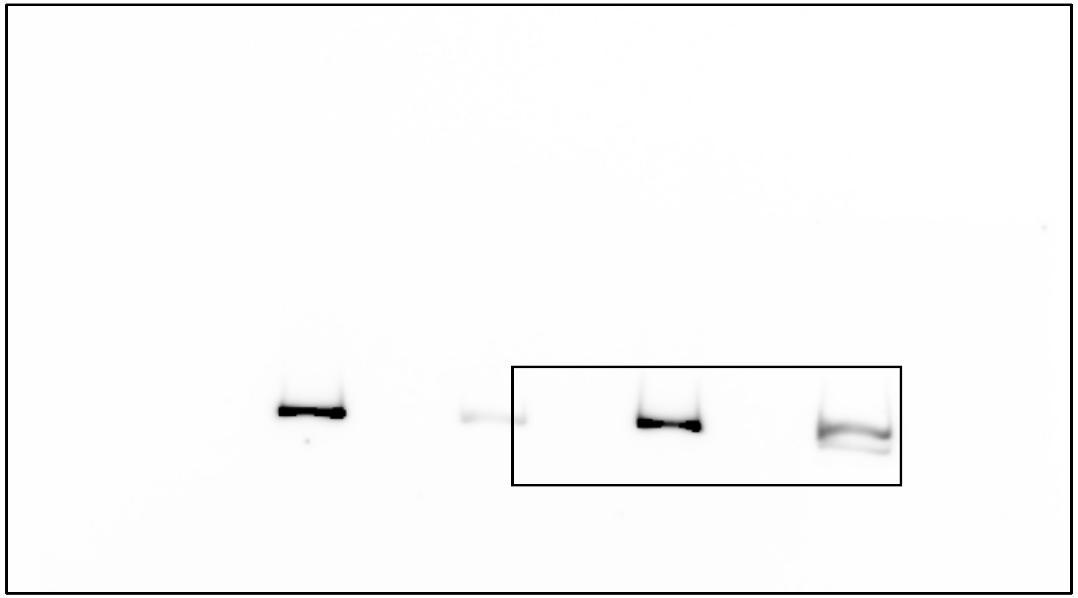

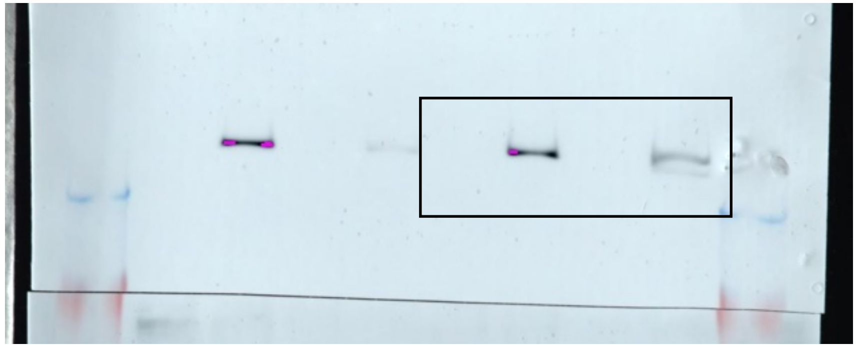

Tubulin

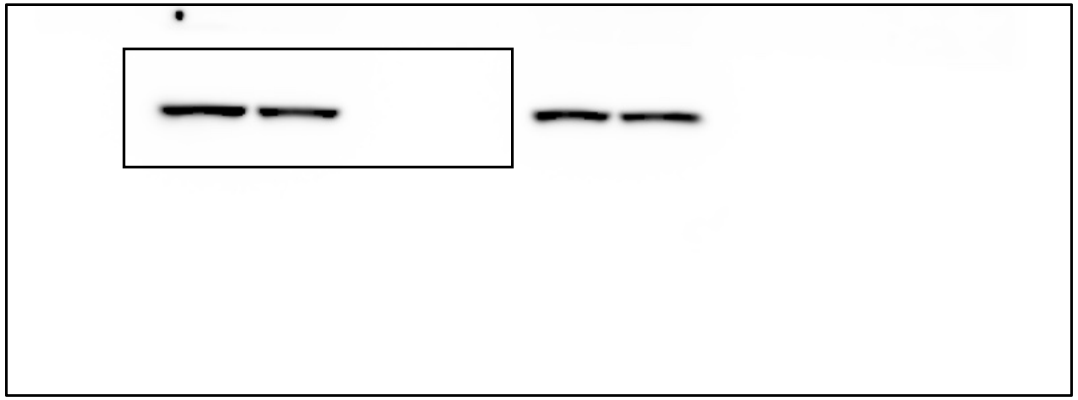

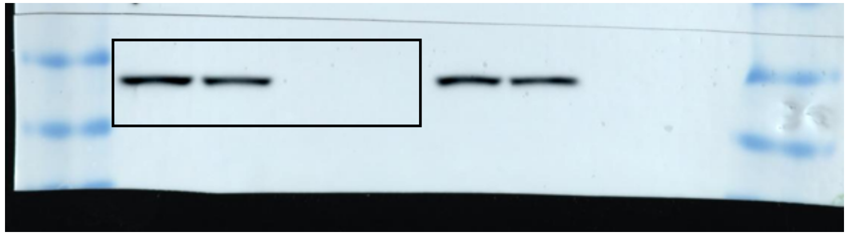

Supplement: cvae121_Supplementary_Data [file cvae121_supplementary_data.zip › Meurs et al source image files (reduced).pdf]
