# Supplementary material for: A suite of genome-engineered hepatic cells provides novel insights into the spatiotemporal metabolism of apolipoprotein B and apolipoprotein B–containing lipoprotein secretion
Source: Cardiovasc Res. 2024 Jun 4;120(11):1253–64. doi: 10.1093/cvr/cvae121 (PMC11416059; doi:10.1093/cvr/cvae121)
Supplement: cvae121_Supplementary_Data [file cvae121_supplementary_data.zip › Meurs et al supp fig legends(modified).docx]

**A suite of genome-engineered hepatic cells provide novel insights into the spatiotemporal metabolism of APOB and APOB-containing lipoprotein secretion**

Amber Meurs^1^, Klevis Ndoj^1^, Marlene van den Berg^1^, Goran Marinković^1,&^, Matteo Tantucci^2^, Tineke Veenendaal^2^, Jan Albert Kuivenhoven^3^, Judith Klumperman^2^, Noam Zelcer^1‡^

^1^ Department of Medical Biochemistry, Amsterdam UMC, Amsterdam Gastroenterology Endocrinology Metabolism and Amsterdam Cardiovascular Sciences, University of Amsterdam, Meibergdreef 9, 1105AZ, Amsterdam, The Netherlands

^2^ Center for Molecular Medicine – Cell Biology, University Medical Center Utrecht, University of Utrecht, Heidelberglaan 100, 3584CX, Utrecht, The Netherlands

^3^ Department of Pediatrics, University Medical Center Groningen, University of Groningen, 9713 AV Groningen, the Netherlands

^&^ Current address: Cerba Research, Visseringlaan 25, 2288ER, Rijswijk, the Netherlands

^‡^ To whom correspondence should be addressed:

Noam Zelcer, PhD

Department of Medical Biochemistry

Academic Medical Center of the University of Amsterdam

Meibergdreef 9

1105AZ Amsterdam

+31-20-5665131

n.zelcer@amsterdamumc.nl

***Running title:*** A suite of hepatic cells to study APOB metabolism and lipoprotein secretion

***Keywords:*** APOB, hepatocytes, MASLD, ERAD, SYVN1, HRD1, VLDL, lipoprotein metabolism

***Supplemental figure legends***

**Supplementary Figure 1. Generation and characterization of Huh7-APOB^mNeon^ cells.** (***A***) Genomic DNA was isolated from HepG2-APOB^mNeon^ and Huh7-APOB^mNeon^ clones and subjected to PCR. Amplification of the wild type and engineered alleles results in a 1024 bp and 2212 bp amplicon, respectively. bp; base pairs. *Note:* Samples were run on the same gel and the dashed line indicates adjoining sections. (***B***) Immunoblot of the indicated proteins in total cell lysate and culture media of Huh-7 cells; Parental cells (WT), ApoB-mNeon-edited cells (KI). (***C***) Intracellular APOB-mNeon in Huh-7 was analyzed by FACS; Parental cells (WT), ApoB-mNeon-edited cells (KI). (N=3). (***B***) Immunoblot is representative of at least 3 independent experiments. (***C***) Box plot shows the mean (middle line), 25th, 75th percentile (box) and minimum and maximum values (whiskers). Data was analyzed using a Student’s t-test. ** p < 0.01, *** p < 0.001.

**Supplementary Figure 2. Proliferation of parental HepG2 and HepG2-APOB^mNeon^ cells is not different.** Parental HepG2 (*i.e.* non genome-edited and clonal selected) and HepG2-APOB^mNeon^ (AM1) cells were seeded and their growth continuously monitored. Proliferation, as evaluated by measurement of confluency was determined at 4 hour intervals. Each point is the mean ± SD of 3 replicates and a representative of 3 independent experiments is shown.

**Supplementary Figure 3. APOB localizes to endocytic compartments.** Immuno-EM. Ultrathin cryosections were immuno-gold labeled using anti-APOB antibody (10 nm gold particles). (***A***) APOB is present at the plasma membrane (P) and occasionally in clathrin coated pits (CCP). (***B***) In addition to the endoplasmic reticulum (ER), APOB is also found in multivesicular endosomes (E). Bars, 100 nm

**Supplementary Figure 4. APOB-mNeon secretion in SYVN-ablated HepG2-APOB^mNeon^ cells.** HepG2-APOB^mNeon^ *AAVS* (control)*, SYVN1*-, and *AMFR-*ablated cells were cultured as described in Figure 6C. Subsequently, culture medium was replaced and secretion of APOB-mNeon into the culture medium was determined after 24 hrs. Box plots show the mean (middle line), 25th, 75th percentile (box) and minimum and maximum values (whiskers).
