# Supplementary material for: A suite of genome-engineered hepatic cells provides novel insights into the spatiotemporal metabolism of apolipoprotein B and apolipoprotein B–containing lipoprotein secretion
Source: Cardiovasc Res. 2024 Jun 4;120(11):1253–64. doi: 10.1093/cvr/cvae121 (PMC11416059; doi:10.1093/cvr/cvae121)
Supplement: cvae121_Supplementary_Data [file cvae121_supplementary_data.zip › Meurs et al Supplemental Table 1 (revision).docx]

**Supplemental Table 1 - List of chemicals.**

| **Chemical name** | **Company** | **Catalog #** |
| --- | --- | --- |
| Poly-L-lysine hydrobromide | Sigma Aldrich | P9155 |
| bovine serum albumin | Sigma Aldrich | A3311 |
| Tunicamycin | Sigma Aldrich | T7765 |
| Paraformaldehyde | Boster | AR1068 |
| CP-346086 | Axon Medchem | Axon 2216 |
| MG132 | Calbiochem | 474790 |
| β-mercaptoethanol | Sigma Aldrich | M6250 |
| ProLong Glass Antifade Mountant | Invitrogen | P36983 |
| UltraPure Agarose | Invitrogen | [16500500](https://www.thermofisher.com/order/catalog/product/16500500) |
| Phusion High Fidelity DNA polymerase | New England Biolabs | M0530L |
| Oleic Acid-Albumin from bovine serum | Sigma Aldrich | O3008 |
| SuperSignal™ West Pico PLUS Chemiluminescent Substrate | Thermo Scientific | 34580 |
