# Supplementary material for: A suite of genome-engineered hepatic cells provides novel insights into the spatiotemporal metabolism of apolipoprotein B and apolipoprotein B–containing lipoprotein secretion
Source: Cardiovasc Res. 2024 Jun 4;120(11):1253–64. doi: 10.1093/cvr/cvae121 (PMC11416059; doi:10.1093/cvr/cvae121)
Supplement: cvae121_Supplementary_Data [file cvae121_supplementary_data.zip › Meurs et al Supplemental Table 2 (revision).docx]

**Supplemental Table 2 - Sequence of homology arms.**

Homology arms used to produce the TVBB-APOB_LH_-mNeonGreen-P2A-Blast-APOB_RH_ plasmid. Sap1 sites are indicated (blue, underlined). The marked T (red, bold) is a silent mutation that was introduced to remove the PAM site used for HDR integration.

Left homology arm fragment:

AACGCTCTTCATACTATTATGAACTTGAAGAAAAGATAGTCAGTCTGATCAAGAACCTGTTAGTTGCTCTTAAGGACTTCCATTCTGAATATATTGTCAGTGCCTCTAACTTTACTTCCCAACTCTCAAGTCAAGTTGAGCAATTTCTGCACAGAAATATTCAGGAATATCTTAGCATCCTTACCGATCCAGATGGAAAAGGGAAAGAGAAGATTGCAGAGCTTTCTGCCACTGCTCAGGAAATAATTAAAAGCCAGGCCATTGCGACGAAGAAAATAATTTCTGATTACCACCAGCAGTTTAGATATAAACTGCAAGATTTTTCAGACCAACTCTCTGATTACTATGAAAAATTTATTGCTGAATCCAAAAGATTGATTGACCTGTCCATTCAAAACTACCACACATTTCTGATATACATCACGGAGTTACTGAAAAAGCTGCAATCAAC**T**ACAGTCATGAACCCCTACATGAAGCTTGCTCCAGGAGAACTTACTATCATCCTCGGCTGAAGAGCGCG

Right homology arm fragment:

CGCGCTCTTCGTGATTTTTTAAAAGAAATCTTCATTTATTCTTCTTTTCCAATTGAACTTTCACATAGCACAGAAAAAATTCAAACTGCCTATATTGATAAAACCATACAGTGAGCCAGCCTTGCAGTAGGCAGTAGACTATAAGCAGAAGCACATATGAACTGGACCTGCACCAAAGCTGGCACCAGGGCTCGGAAGGTCTCTGAACTCAGAAGGATGGCATTTTTTGCAAGTTAAAGAAAATCAGGATCTGAGTTATTTTGCTAAACTTGGGGGAGGAGGAACAAATAAATGGAGTCTTTATTGTGTATCATACCACTGAATGTGGCTCATTTGTATTGAAAGACAGTGAAACGAGGGCATTGATAAAATGTTCTGGCACAGCAAAACCTCTAGAACACATAGTGTGATTTAAGTAACAGAATAAAAATGGAAACGGAGAAATTATGGAGGGAAATATTTTGCAAAAATATTTAAAAAGATGAGGTAATTGTGTTTTTATAATCGAAGAGCGTT
