# Supplementary material for: A suite of genome-engineered hepatic cells provides novel insights into the spatiotemporal metabolism of apolipoprotein B and apolipoprotein B–containing lipoprotein secretion
Source: Cardiovasc Res. 2024 Jun 4;120(11):1253–64. doi: 10.1093/cvr/cvae121 (PMC11416059; doi:10.1093/cvr/cvae121)
Supplement: cvae121_Supplementary_Data [file cvae121_supplementary_data.zip › Meurs et al Supplemental Table 3 (revision).docx]

**Supplemental Table 3 - List of antibodies**

| **Target protein** | **Company** | **Catalogus nr.** | **Immunoblot** | **Immuno Fluorescence** |
| --- | --- | --- | --- | --- |
| APOB | Proteintech | 20578-1-AP | 1:1,000 |  |
| APOB | Calbiochem | 178467 |  | (**EM**) 1:3000 |
| mNeonGreen | Chromotek | 32f6-100 | 1:1,000 |  |
| Calnexin | Sigma | C4731 | 1:5,000 | 1:200 |
| Tubulin | Sigma | T9026 | 1:5,000 |  |
| Goat Anti-Rabbit IgG - HRP superclonal | Life Technologies | A27036 | 1:10,000 |  |
| Goat Anti-Mouse IgG (H+L) - HRP | Life Technologies | A28177 | 1:10,000 |  |
| GM130 | Cell signaling | 12480 |  | 1:200 |
| Donkey anti-Rabbit IgG (H+L) Secondary Antibody, Alexa Fluor 568 | Thermo Fischer | A10042 |  | 1:250 |
