# Supplementary material for: A suite of genome-engineered hepatic cells provides novel insights into the spatiotemporal metabolism of apolipoprotein B and apolipoprotein B–containing lipoprotein secretion
Source: Cardiovasc Res. 2024 Jun 4;120(11):1253–64. doi: 10.1093/cvr/cvae121 (PMC11416059; doi:10.1093/cvr/cvae121)
Supplement: cvae121_Supplementary_Data [file cvae121_supplementary_data.zip › Meurs et al Supplemental Table 4 (revision).docx]

**Supplemental Table 4 - Sequence of sgRNAs**

| **Target gene** | **Forward Primer** | **Reverse Primer** |
| --- | --- | --- |
| APOB #1 | CACCGAGTTGTATGTGTACTTCCGG | AAACCCGGAAGTACACATACAACTC |
| APOB #2 | CACCGGGTGTATGGCTTCAACCCTG | AAACCAGGGTTGAAGCCATACACCC |
| MTTP #1 | CACCGAAAGGGGAGGGGTTTATGCG | AAACCGCATAAACCCCTCCCCTTTC |
| MTTP #2 | CACCGGGAAAACTGCAAGACAGCGT | AAACACGCTGTCTTGCAGTTTTCCC |
| AAVS #1 | CACCGGTTAATGTGGCTCTGGTTCT | AAACAGAACCAGAGCCACATTAACC |
| AMFR #1 | CACCGACAGGGACAGGACTCGACCG | AAACCGGTCGAGTCCTGTCCCTGTC |
| AMFR #2 | CACCGCACAAGAACTATCTACGTG | AAACCACGTAGATAGTTCTTGTGC |
| AMFR #3 | CACCGCCTCAACCACGAAGGGACGT | AAACACGTCCCTTCGTGGTTGAGGC |
| CGRRF #1 | CACCGACCCCCAGTAGCATGTAAGG | AAACCCTTACATGCTACTGGGGGTC |
| CGRRF #2 | CACCGAAGGTCAATAGCGCTACCAA | AAACTTGGTAGCGCTATTGACCTTC |
| CGRRF #3 | CACCGACACTTACCATCCCAATACC | AAACGGTATTGGGATGGTAAGTGTC |
| MARCH8 #1 | CACCGCTCATTGACCGTACTGCTG | AAACCAGCAGTACGGTCAATGAGC |
| MARCH8 #2 | CACCGTTCTATCACGCCATCCAGCC | AAACGGCTGGATGGCGTGATAGAAC |
| MARCH8 #3 | CACCGCTGCAAGTATGAGTTCATCA | AAACTGATGAACTCATACTTGCAGC |
| MARCH6 #1 | CACCGCCCCACCGTTCAATGCTGCG | AAACCGCAGCATTGAACGGTGGGGC |
| MARCH6 #2 | CACCGTCGAGAACTGAGCTTTCAGT | AAACACTGAAAGCTCAGTTCTCGAC |
| MARCH6 #3 | CACCGTACTCGAACAGGGACACACG | AAACCGTGTGTCCCTGTTCGAGTAC |
| ZNRF4 #1 | CACCGTTGAGTTGGAATGACCGCG | AAACCGCGGTCATTCCAACTCAAC |
| ZNRF4 #2 | CACCGCCTCGCTCACGAACACTGAG | AAACCTCAGTGTTCGTGAGCGAGGC |
| ZNRF4 #3 | CACCGCGAAGGTGCAGTCGTAGCGG | AAACCCGCTACGACTGCACCTTCGC |
| RNFT1 #1 | CACCGTGTGTACACAGTCGCTTACG | AAACCGTAAGCGACTGTGTACACAC |
| RNFT1 #2 | CACCGAAGCTCAAATGGTCCAAAGT | AAACTCTTTGGACCATTTGAGCTTC |
| RNFT1 #3 | CACCGAATCTTGTGTGGACACACTG | AAACCAGTGTGTCCACACAAGATTC |
| TRIM59 #1 | CACCGGTAAAGATTCAATGCCAGT | AAACACTGGCATTGAATCTTTACC |
| TRIM59 #2 | CACCGTGTGGTCATTGCCTTACCAT | AAACATGGTAAGGCAATGACCACAC |
| TRIM59 #3 | CACCGCAACATCACAGAGAGCCGTT | AAACAACGGCTCTCTGTGATGTTGC |
| RNF128 #1 | CACCGCACACGGGAGTGAACCGTA | AAACTACGGTTCACTCCCGTGTGC |
| RNF128 #2 | CACCGTGGTTGGCCCTCATCCAACG | AAACCGTTGGATGAGGGCCAACCAC |
| RNF128 #3 | CACCGATTCTGCAATCTATTCAAAG | AAACCTTTGAATAGATTGCAGAATC |
| RNF13 #1 | CACCGGAATTAGGTAGTATTCCAA | AAACTTGGAATACTACCTAATTCC |
| RNF13 #2 | CACCGCGTTGGATCCCATGCTAATG | AAACCATTAGCATGGGATCCAACGC |
| RNF13 #3 | CACCGTGGAGGCACTATGGGTTCAC | AAACGTGAACCCATAGTGCCACCAC |
| RNF139 #1 | CACCGTTAATGTACATCTTAAGGA | AAACTCCTTAAGATGTACATTAAC |
| RNF139 #2 | CACCGCACTGAGAGACATTAATACA | AAACTGTATTAATGTCTCTCAGTGC |
| RNF139 #3 | CACCGATTCTACACTAACTGTACT | AAACAGTACAGTTAGTGTAGAATC |
| RNF148 #1 | CACCGTGACAGTCATCATTGAAGTG | AAACCACTTCAATGATGACTGTCAC |
| RNF148 #2 | CACCGGTTGGAAATGAGATCACAT | AAACATGTGATCTCATTTCCAACC |
| RNF148 #3 | CACCGCTGGCCCTCATCGAACGTGG | AAACCCACGTTCGATGAGGGCCAGC |
| RNF150 #1 | CACCGCTCTGCCTGGACTTTACCG | AAACCGGTAAAGTCCAGGCAGAGC |
| RNF150 #2 | CACCGAGACAAACACAACCGAAGTG | AAACCACTTCGGTTGTGTTTGTCTC |
| RNF150 #3 | CACCGCAAGGGCAACTGCACGTACA | AAACTGTACGTGCAGTTGCCCTTGC |
| RNF170 #1 | CACCGTTCCCGGTGGAGACCAACTG | AAACCAGTTGGTCTCCACCGGGAAC |
| RNF170 #2 | CACCGCGATATGGTTCATGGCTTG | AAACCAAGCCATGAACCATATCGC |
| RNF170 #3 | CACCGAGTGTAGAACTGCTGTCGAG | AAACCTCGACAGCAGTTCTACACTC |
| MARCH2 #1 | CACCGCTCCAGACAGCTCTTATGCA | AAACTGCATAAGAGCTGTCTGGAGC |
| MARCH2 #2 | CACCGCTGCCGGAGCAGTCACACA | AAACTGTGTGACTGCTCCGGCAGC |
| MARCH2 #3 | CACCGTCCAAGGCACGGATGACGG | AAACCCGTCATCCGTGCCTTGGAC |
| MARCH3 #1 | CACCGCAAACCCAGGCCGTTAGTGG | AAACCCACTAACGGCCTGGGTTTGC |
| MARCH3 #2 | CACCGAAACTTGCATGACATACTG | AAACCAGTATGTCATGCAAGTTTC |
| MARCH3 #3 | CACCGACATCGGCCGGTCATTGAAG | AAACCTTCAATGACCGGCCGATGTC |
| MARCH4 #1 | CACCGACTTGACCGAGCCATCACAG | AAACCTGTGATGGCTCGGTCAAGTC |
| MARCH4 #2 | CACCGTTGTTGGCCGCCAAACCGGG | AAACCCCGGTTTGGCGGCCAACAAC |
| MARCH4 #3 | CACCGACCGAGGATCGCTACTCACT | AAACAGTGAGTAGCGATCCTCGGTC |
| RNF185 #1 | CACCGCTGAGAACTCCAGTGCAGGG | AAACCCCTGCACTGGAGTTCTCAGC |
| RNF185 #2 | CACCGGAGACCAGACCTAACAGAC | AAACGTCTGTTAGGTCTGGTCTCC |
| RNF185 #3 | CACCGCATCTTACCTGATGTAAACA | AAACTGTTTACATCAGGTAAGATGC |
| RNF26 #1 | CACCGAGACTAGTCAGTACCGTGTG | AAACCACACGGTACTGACTAGTCTC |
| RNF26 #2 | CACCGAGCAGGGAGATACTGCACCG | AAACCGGTGCAGTATCTCCCTGCTC |
| RNF26 #3 | CACCGCCCACATGTGAACCGGACCA | AAACTGGTCCGGTTCACATGTGGGC |
| BFAR #1 | CACCGTGACACGTTCTAGCTCCATG | AAACCATGGAGCTAGAACGTGTCAC |
| BFAR #2 | CACCGGCTGTGGCCAAATGGACGG | AAACCCGTCCATTTGGCCACAGCC |
| BFAR #3 | CACCGATTCCTTTAGCTCCTAACAC | AAACGTGTTAGGAGCTAAAGGAATC |
| RNF5 #1 | CACCGCTCGCGATTTGGCCCTTCG | AAACCGAAGGGCCAAATCGCGAGC |
| RNF5 #2 | CACCGACCAAATGGCTGGAATCCCT | AAACAGGGATTCCAGCCATTTGGTC |
| RNF5 #3 | CACCGTCGATTCTCACCAGTACAGG | AAACCCTGTACTGGTGAGAATCGAC |
| TRIM13 #1 | CACCGCAGTATGCAAAGGACACTTG | AAACCAAGTGTCCTTTGCATACTGC |
| TRIM13 #2 | CACCGCAAGTGTCCTACATGCCGTA | AAACTACGGCATGTAGGACACTTGC |
| TRIM13 #3 | CACCGATACCTTGGAAACTAGTAAG | AAACCTTACTAGTTTCCAAGGTATC |
| SYVN1 #1 | CACCGGCCTGGATGTACAGGACCT | AAACAGGTCCTGTACATCCAGGCC |
| SYVN1 #2 | CACCGTCAGGATGCTGTGATAGGCG | AAACCGCCTATCACAGCATCCTGAC |
| SYVN1 #3 | CACCGCCTCCAGAGTGAGAACCCCT | AAACAGGGGTTCTCACTCTGGAGGC |
| RNF145 #1 | CACCGACTGGAGGCAGTGTTAAATG | AAACCATTTAACACTGCCTCCAGTC |
| RNF145 #2 | CACCGGGACATTCCCAAGGCGAGA | AAACTCTCGCCTTGGGAATGTCCC |
| RNF145 #3 | CACCGATCAGCCTGCATCACGTGAG | AAACCTCACGTGATGCAGGCTGATC |
| RNF103 #1 | CACCGCACCGGATCCACCAGCTGGG | AAACCCCAGCTGGTGGATCCGGTGC |
| RNF103 #2 | CACCGAGCTGTAAAAAGCCCAACAC | AAACGTGTTGGGCTTTTTACAGCTC |
| RNF103 #3 | CACCGCAATTAGTAAACCATGACCA | AAACTGGTCATGGTTTACTAATTGC |
