# Supplementary figures and images for: A suite of genome-engineered hepatic cells provides novel insights into the spatiotemporal metabolism of apolipoprotein B and apolipoprotein B–containing lipoprotein secretion
Source: Cardiovasc Res. 2024 Jun 4;120(11):1253–64. doi: 10.1093/cvr/cvae121 (PMC11416059; doi:10.1093/cvr/cvae121)

Supplemental Figure 1

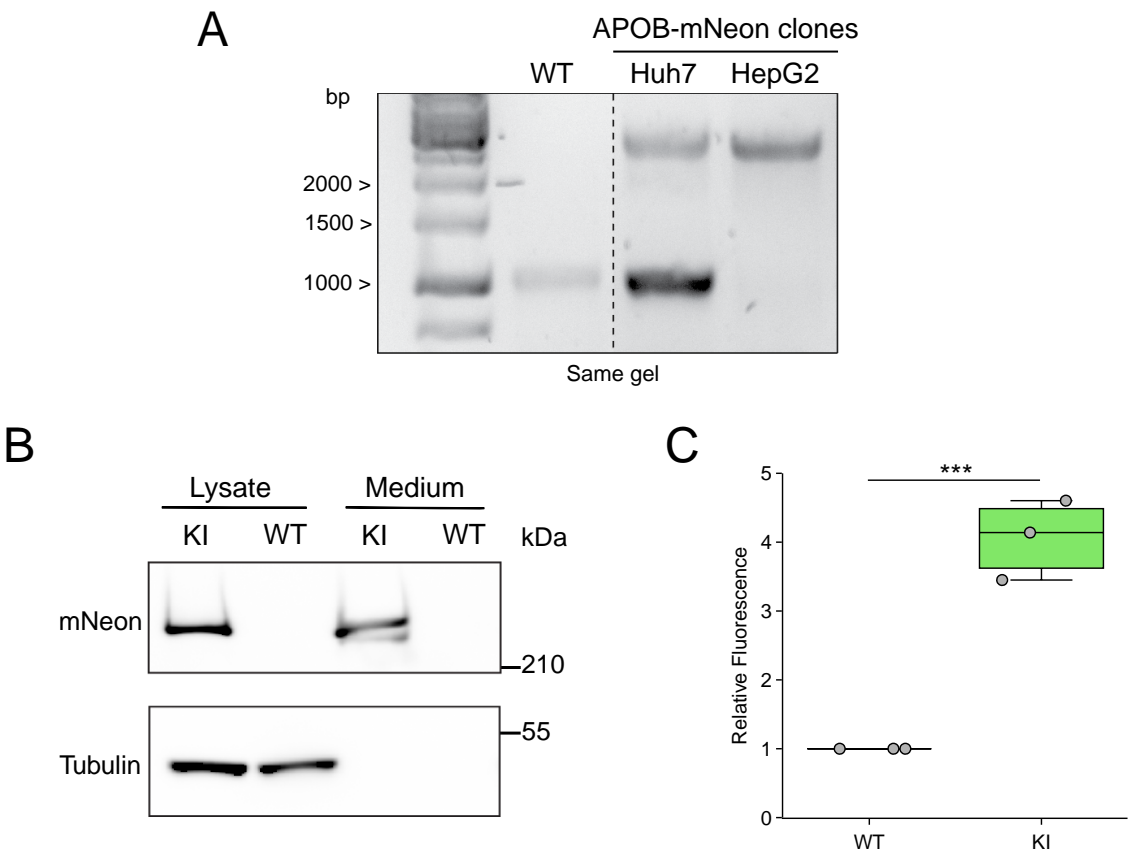

Supplement: cvae121_Supplementary_Data [file cvae121_supplementary_data.zip › Meurs et al Supplemental Figure 1 (modified).pdf]

Supplemental Figure 2

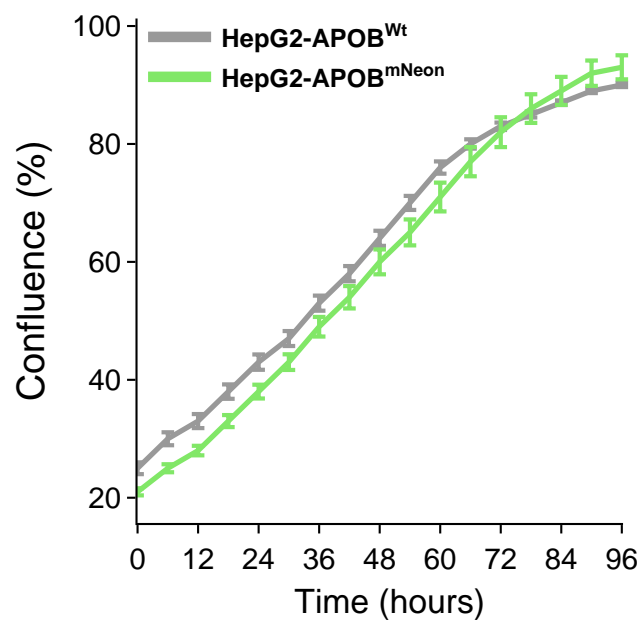

Supplement: cvae121_Supplementary_Data [file cvae121_supplementary_data.zip › Meurs et al Supplemental Figure 2 (revision).pdf]

***Supplemental Figure 3***

**A**

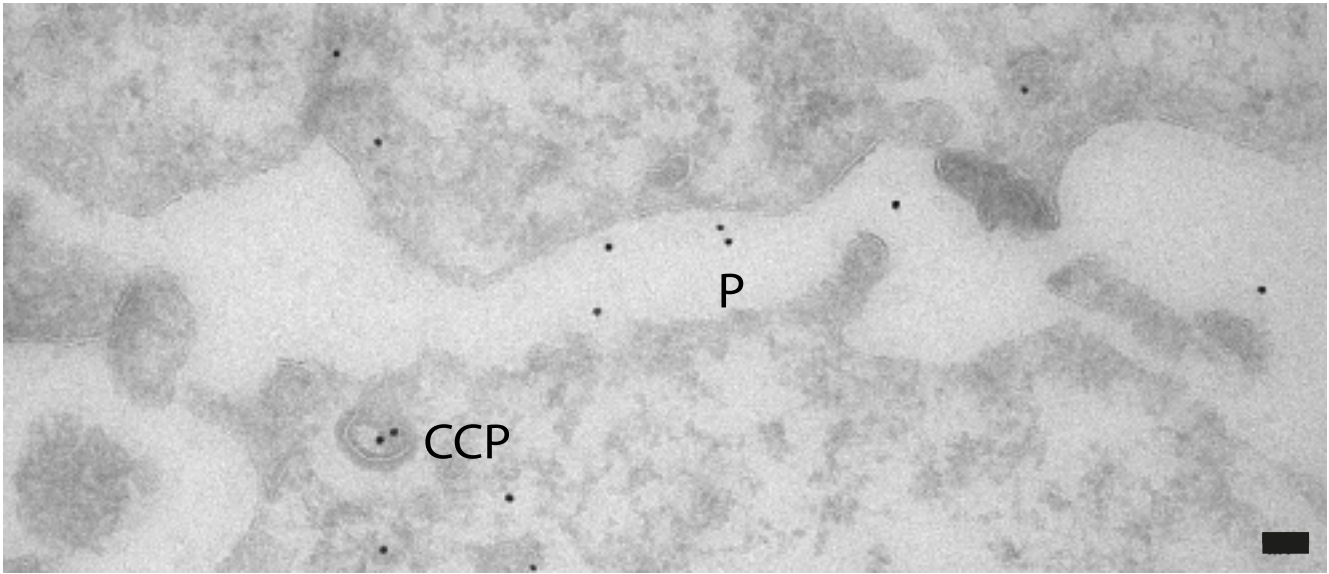

**B**

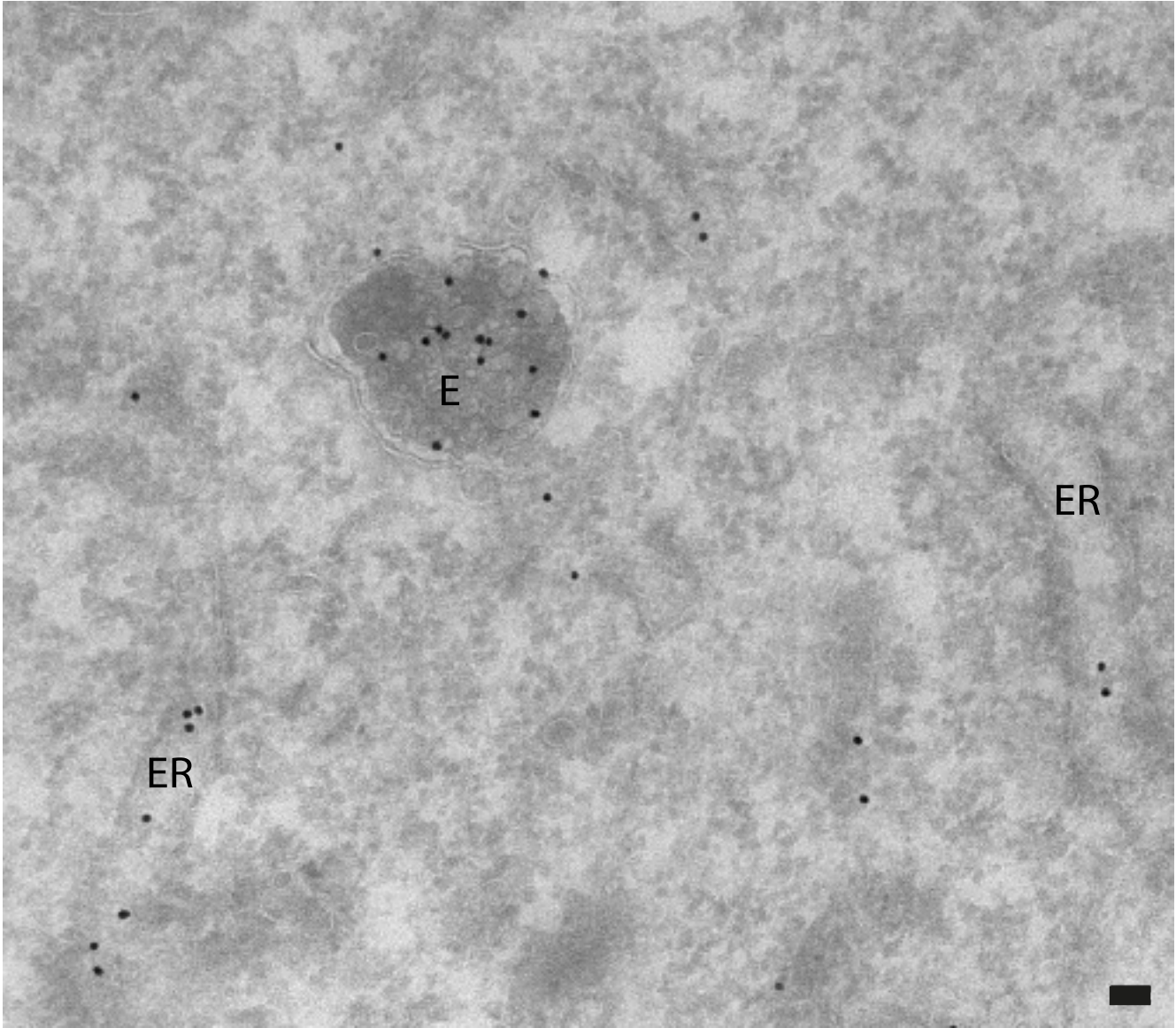

Supplement: cvae121_Supplementary_Data [file cvae121_supplementary_data.zip › Meurs et al Supplemental Figure 3 (revision).pdf]

Supplemental Figure 4

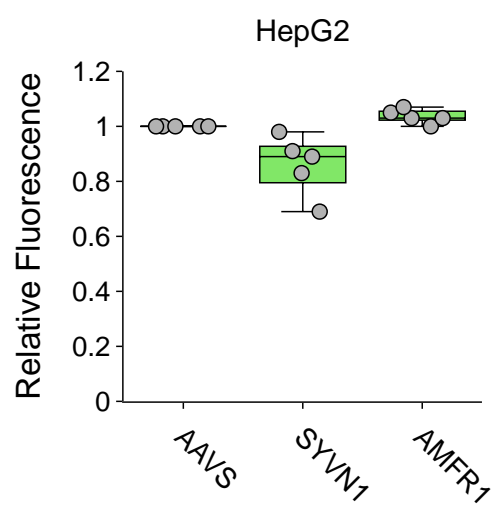

Supplement: cvae121_Supplementary_Data [file cvae121_supplementary_data.zip › Meurs et al Supplemental Figure 4 (revision).pdf]
